# Impact of Small-Alkane Solvents on Polyolefin Hydrogenolysis over a Ruthenium Catalyst

**DOI:** 10.1021/acs.iecr.6c00853

**Published:** 2026-04-30

**Authors:** Pavel A. Kots, Zachary R. Hinton, Mehdi Zare, Brandon C. Vance, María Ley-Flores, Juan J. de Pablo, Thomas H. Epps, LaShanda T. J. Korley, Michele Valsecchi, George Jackson, Amparo Galindo, Dionisios G. Vlachos

**Affiliations:** † Center for Plastic Innovation, 5972 University of Delaware, 221 Academy Street, Newark, Delaware 19716, United States; ‡ Department of Chemical and Biomolecular Engineering, Tandon School of Engineering, New York University, Brooklyn, New York 11201, United States; § Department of Chemical and Biomolecular Engineering, University of Delaware, 150 Academy Street, Newark, Delaware 19716, United States; ∥ Pritzker School of Molecular Engineering, 2462 The University of Chicago, Chicago, Illinois 60637, United States; ⊥ Center for Molecular Engineering, Argonne National Laboratory, Lemont, Illinois 60439, United States; # Department of Materials Science and Engineering and Center for Research in Soft Matter and Polymers (CRiSP), University of Delaware, Newark, Delaware 19716, United States; 7 Department of Chemical Engineering, Sargent Centre for Process Systems Engineering, Institute for Molecular Science and Engineering, Imperial College, South Kensington Campus, London SW7 2AZ, United Kingdom

## Abstract

Selective catalytic hydrogenolysis of polyolefins is a promising route to convert plastic waste into valuable liquid products, such as lubricants, waxes, and surfactants. However, the high viscosity of polymer melts imposes mass transfer limitations on this reaction. Solvents can mitigate these challenges, but their effects on reaction kinetics and product selectivity remain underexplored. Here, we systematically explore the effects of small *n*-alkanes and cycloalkanes on the hydrogenolysis of polyethylene and polypropylene over a Ru/TiO_2_ catalyst. Using kinetic measurements and isotopic labeling, we show that *n*-octane at high mass fractions alters the mechanism from direct hydrogenation to solvent-mediated hydrogen transfer, reducing the rate of C–C bond cleavage. Longer alkanes further inhibit reactivity due to stronger surface binding. 1,4-Dimethylcyclohexane suppresses methane formation, favoring heavier products, while decalin likely forms surface-bound aromatics that poison the catalyst. Overall, alkane solvents modulate product selectivity and reduce the yield of methane byproduct, allowing for ∼35–40% selectivity to valuable C_20_-C_30_ alkane products. This work highlights the complex impact of polymer–alkane mixtures on hydrogenolysis kinetics relevant to the design of commercial-scale plastic waste valorization processes.

## Graphical abstract

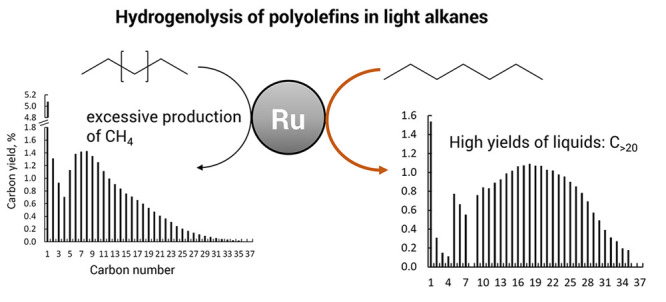

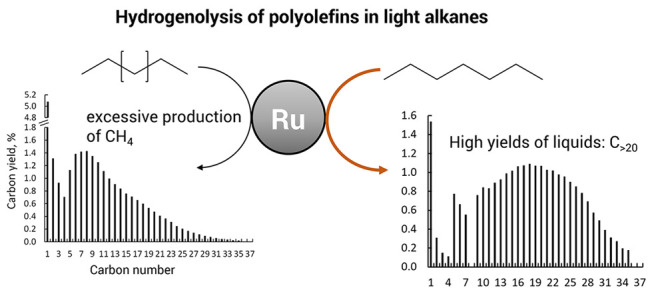

## 1 Introduction

Plastics waste (PW) generation increases with plastic production, leading to its growing accumulation in the environment.^1–3^ Polyolefins (PO) are a major fraction of PW and are often the most challenging to valorize.^4^ A proposed PW management strategy involves the open-loop upcycling of PO to value-added products, such as commodity chemicals.^5,6^ Recently, conventional petrochemical technologies, such as catalytic pyrolysis,^7,8^ hydrocracking,^9^ reforming,^10^ metathesis,^11,12^ and hydrogenolysis,^13,14^ have been adapted to convert POs using solid catalysts, typically operating in polymer melts.

Specifically, hydrogenolysis over supported Ru, Ni, Co, and Pt catalysts effectively converts PO into wax, lubricants, naphtha-range alkanes, and light gases (e.g., methane).^15–17^ This reaction involves C–C bond breaking in the polymer backbone under high-pressure H_2_, similar to small alkanes.^18^ Recent advancements have replaced expensive, precious metal catalysts with less expensive earth-abundant metals such as Ni.^19,20^ Tuning the catalyst structure using metal–support interactions,^13^ coating metal particles with a mesoporous SiO_2_ shell,^21^ mild support reduction,^19^ and other approaches have reduced the required reaction time and fine-tuned the product distribution. Mechanistic studies indicate the crucial role of surface hydrogen coverage (θ_H_) in facilitating the rapid desorption of medium-sized reaction products to avoid secondary hydrogenolysis into light gases.^22,23^

Catalytic reactions in polymer melts are often limited by mass transfer, particularly in scaled-up batch reactors, due to the high viscosity and low diffusion coefficients of the polymer melt/chains.^24,25^ The low thermal conductivity of PO melts results in hot spots, reduced selectivity, and poor overall process control.^26^ Inert solvents can mitigate these challenges by improving convection and mixing and enhancing polymer diffusion; normal and cyclic alkanes can be used for this purpose.^27^ For example, mixing HDPE with a base oil results in a linear decrease in viscosity as a function of the solvent mass fraction.^28^ Conveniently, PO hydrogenolysis produces a mixture of alkanes that can be used as solvents.

Notably, many solvents can alter polymer conformation, block polymer adsorption, or poison the catalyst active sites.^29–31^ Jia et al. proposed, among other factors, that solvent-induced disentanglement of polyethylene chains leads to reduced polymer adsorption on the catalyst surface. Less favorable polymer–solvent interactions result in more compact solvated polymer conformation, leading to a higher adsorption propensity onto the Ru/C surface.^30^ Solvents can also interfere with hydrogen chemisorption and alter θ_H_ on metal surfaces. Ultimately, the knowledge gap related to the solvent effect in polymer deconstruction hinders further implementation and scale-up of solvent-assisted PO upcycling.

Herein, we illustrate the impact of alkane solvents on PO hydrogenolysis over a well-studied Ru/TiO_2_ catalyst. We elucidate how small alkanes as solvents alter intrinsic polymer reactivity, the availability of surface hydrogen, catalyst activity, and reaction selectivity. We also highlight the effect of the solvent on the product selectivity and overall yields. Our approach is extended to low- and high-density polyethylenes of various molecular weights and isotactic polypropylene using a range of common small alkane solvents. Overall, this work addresses the critical role of the solvent environment in PW hydrogenolysis.

## 2 Results and Discussion

### 2.1 Impact of Small-Alkane Solvents on Intrinsic Polymer Reactivity

High-density polyethylene (HDPE) conversion over Ru/TiO_2_ was investigated in a batch reactor under standard hydrogenolysis conditions (250 °C, 30 bar H_2_). At a short reaction time of 0.17 h (10 min), the carbon-atom-based yield of liquid products comprised ∼2% (Table S1), with the remaining major portion consisting of solid product and unreacted polymer. Parallel experiments were conducted, wherein HDPE was premixed with *n*-octane at a mass fraction in the melt phase, ω­(octane), of 0.02–0.4, corresponding to an *n*-octane/HDPE carbon atomic ratio of 0 to 0.65. The amount of octane that formed saturated vapor in the reactor headspace was subtracted to account only for the octane present in the HDPE-octane melt phase. Octane conversion was negligible due to the short reaction time (10 min).

Gel permeation chromatography (GPC) of the solid residue enables the differentiation of unreacted HDPE from hydrogenolysis products using deconvolution (Figures 1a,b and S1).^32^ The solvent-free HDPE fractional conversion was 0.35. The conversion decreased significantly with adding *n*-octane up to ω­(octane) ≈ 0.1. Upon further increasing the octane fraction, conversion reached 0.23, representing 70% of the initial octane-free conversion value, and then remained constant. Clearly, the effect of *n*-octane addition is nonlinear (Figure 1b).

**Figure 1:**
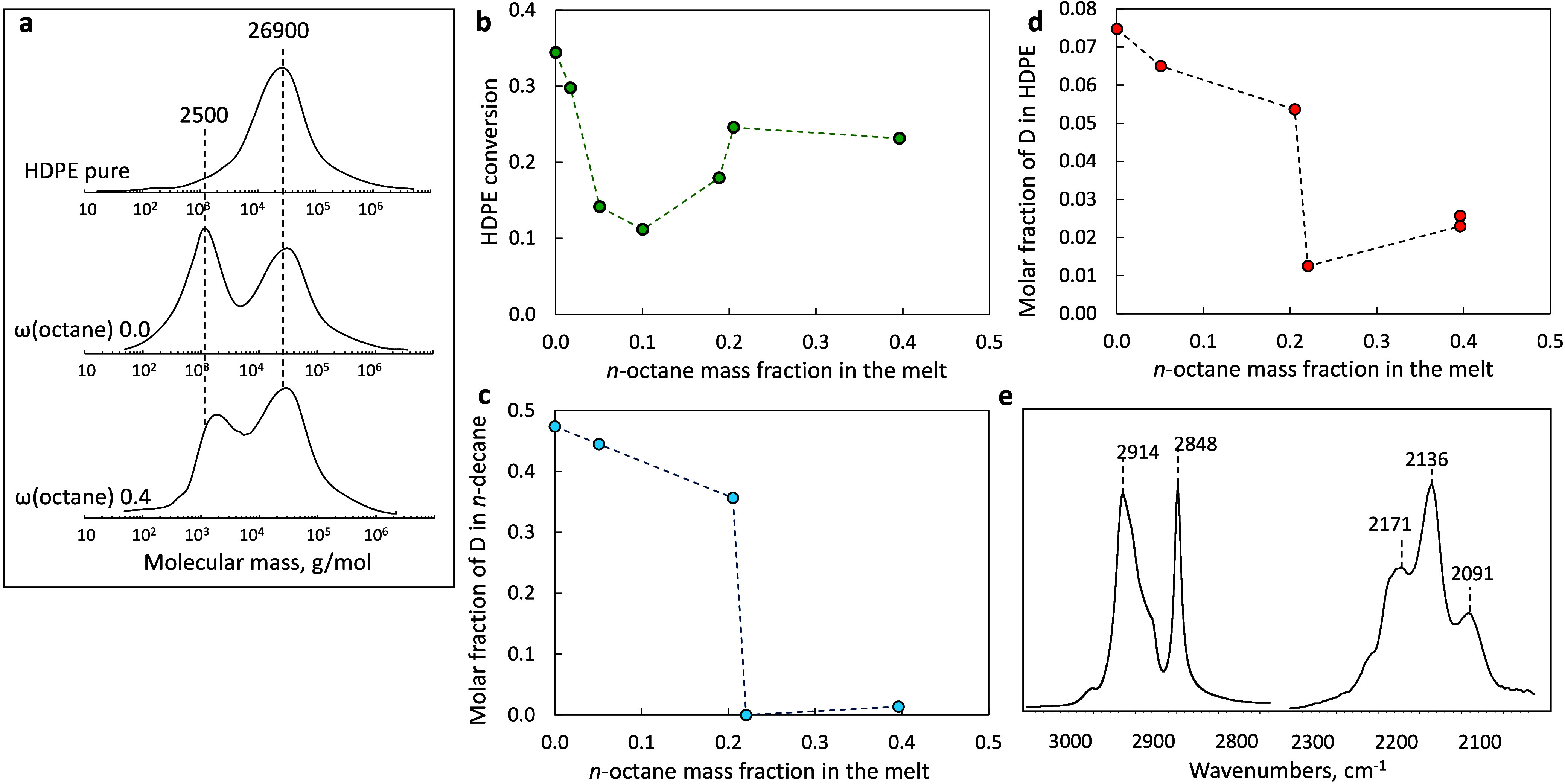
(a) Molar mass distributions (measured via GPC) for pristine HDPE and solid residue mixtures. (b) HDPE conversion as a function of the initial *n*-octane mass fraction. (c, d) Molar fraction of D atoms in *n*-decane (reaction product) and residual solid at different initial *n*-octane mass fractions. (e) Attenuated total reflectance (ATR) Fourier transform infrared (FTIR) spectrum of a deuterated solid residue. Conditions: 250 °C, 30 bar H_2_ (or D_2_), reaction time 10 min, 2 g of HDPE, and 50 mg of Ru/TiO_2_ catalyst.

The number-average molecular weight (*M*_n_) of the reaction product is correlated with conversion—a higher conversion leads to a lighter product (Table S2). Hydrogenolysis kinetics were semiquantitatively analyzed to elucidate the rates of C–C bond scission in HDPE. Table S2 shows that apparent rates (*r*_p_) decreased upon the addition of octane. Given highly probable zeroth-order kinetics for HDPE hydrogenolysis over the Ru catalyst,^33,34^ one can expect a similar decline in apparent rate constants. The strong dependence of HDPE conversion and *r*_p_ on ω­(octane) may arise from several factors, as discussed below.

#### 2.1.1 Hydrogen Concentration

If hydrogen chemisorption is at equilibrium, then the solvent cannot alter the hydrogen chemical potential in the polymer melt, which is set by the H_2_ partial pressure in the reactor headspace. However, we hypothesize that adding *n*-octane to the HDPE melt increases the concentration of dissolved H_2_ (*c*_H_) in the melt. Under nonequilibrium conditions, a higher *c*_H_ can increase the hydrogen chemisorption and hydrogen coverage (*θ*_H_) on Ru and increase the reaction rate.^22^ Calculations show that adding *n*-octane can increase the dissolved hydrogen concentration by 10–20% (Figure S3). As a result, the *n*-octane addition is expected to enhance the hydrogen solubility and the hydrogenolysis reaction rate, which is not observed experimentally.

Following the analysis of Ge and Peters,^24^ we estimate the Henry constant for H_2_ absorption in bulk HDPE (solvent-free case) as 3.9 × 10^–3^ mol_H_2__·L^–1^·bar^–1^ at 250 °C, leading to a relatively high *c*_H_ ≈ 0.2 mol_H_2__·L^–1^. Extrapolation from literature data^35^ for pure *n*-octane shows *c*_H_ ≈ 0.27 mol_H_2__·L^–1^, slightly higher than for pure HDPE and consistent with our estimates (Figure S3).

According to Jaydev et al., hydrogen mass transfer from the gas phase to the melt can potentially limit the overall reaction rate;^25^ however, it is unlikely that the addition of *n*-octane will substantially change the hydrogen availability in the melt since it does not substantially alter *c*_H_ in the liquid phase.

#### 2.1.2 Phase Segregation

The phase segregation of HDPE and *n*-octane may decrease the catalyst activity. Calculations with the SAFT-γ Mie equation indicate no phase separation between octane and HDPE under our experimental conditions, consistent with the literature.^36^ The characteristic diffusion length *L*_D_^HDPE^ of the polymer is

1 $$L_{D}^{HDPE}\approx \sqrt{\tau D_{s}}\approx \sqrt{600s\times 9.8\times 10^{-10}\frac{cm^{2}}{s}}\approx 7.7\mu m$$

wherein τ is the reaction time (10 min) and *D*_s_ is the self-diffusion coefficient.^37^ The polymer mobility within 10 min at 250 °C is on the micrometer scale, indicating that it is relatively stagnant. Octane’s diffusion distance is *L*_D_^octane^ ≈ 1 mm due to the polymer’s diffusion being ∼4 to 5 orders of magnitude slower than *n*-octane’s. The high *L*_D_^octane^ indicates that *n*-octane would penetrate the polymer melt upon contact under the reaction conditions. Thus, despite the polymer’s low mobility, *n*-octane-HDPE segregation can be ruled out from both the mass transfer and thermodynamics perspectives.

#### 2.1.3 Polymer–Octane Interaction in the Bulk

The presence of *n*-octane in the polymer melt reduces the concentration of chain entanglements in the mixture, increasing chain mobility and polymer entropy in the melt.^30^ The entropy loss associated with polymer adsorption on Ru would limit polymer–catalyst binding, favoring polymer–solvent interactions. N-Octane is expected to behave as a good solvent and suppress polymer adsorption, with a Flory–Huggins interaction parameter of χ < 0.5.^38^ Thus, the adsorption of *n*-octane would be preferred over polymer due to the high entropic penalty of polymer immobilization on the surface. More detailed thermodynamics-based delineation between bulk and surface interactions is given in Supplementary Discussion I.

A reduction in HDPE conversion occurs at low ω­(octane) < 0.1; octane would not substantially disrupt entanglements in the melt at such a low concentration.^37^ The minor impact of octane on the chain’s mobility is also visible in Figure S5, with *D*_s_ plotted as a function of ω­(octane). Significant diffusivity enhancement, connected to higher entanglement mobility, occurs at much higher *n*-octane concentrations. Nonetheless, we cannot completely rule out that at short reaction times the disengagement can impact the apparent reaction rate. On the other hand, our data in Section 3, using hexadecane, tetracosane (C_24_H_50_), and decalin at longer reaction times (∼0.5–2 h), reveal that catalyst activity does not correlate with polymer–solvent interactions.

#### 2.1.4 Mixing within the Melt

Catalyst activity is partially recovered at high ω­(octane) due to improved mixing and faster mass transfer (Figure 1b). The HDPE self-diffusivity (*D*_s_) in a mixture with *n*-octane increases from 9.8 × 10^–10^ to 7.3 × 10^–9^ cm^2^·s^–1^ when ω­(octane) increases from 0 to 0.4 at 250 °C (Figure S5).^37^ Molecular dynamic simulations show a high diffusion coefficient of octane of ∼3 × 10^–5^ cm^2^·s^–1^ in the same composition range. Because the polymer self-diffusion correlates with the melt absolute viscosity (η), η ≈ *D*_s_^–1.84^,^39^ we expect the viscosity to drop from ∼10^2^ to ∼10^0^ Pa·s at ω­(octane) = 0.4. Experimental data on an HDPE/C_30_H_62_ wax blend shows η ≈ 10^1^ Pa·s in a similar concentration range.^28^ Octane–HDPE mixtures in an ω­(octane) range of 0.3–0.4 are very close to the operational range of laboratory magnetic stirrers. Control experiments in a glass flask reveal that adding hexadecane increases melt flow (i.e., reduces viscosity), resulting in significantly faster mixing with a magnetic stirrer. Therefore, the recovery of HDPE conversion at high octane concentrations (Figure 1b) is likely related to the improved mixing within the reaction mixture.

#### 2.1.5 Polymer-Ru/TiO_2_ Interactions

Adding a small alkane could affect the polymer adsorption on the catalyst.^29^ We propose that the competitive adsorption of octane over polymer leads to a decline in HDPE conversion in the ω­(octane) range of 0.02–0.1. To demonstrate this effect, we performed H/D exchange to characterize polymer–Ru/TiO_2_ interactions. Experiments with D_2_ instead of H_2_ showed a substantial deuteration of all liquid reaction products. *n*-Decane was selected from the product mixture as a typical example; however, other liquid products exhibit similar behavior (Figures 1c and S6). Without octane addition, *n*-decane produced from the D_2_/HDPE mixture has a 47% deuteration degree and a mean composition of C_10_H_11.66_D_10.34_. The addition of octane reduces deuteration linearly, with an abrupt decline at ω­(octane) ≈ 0.21, from 36 to 1% D. A similar trend is observed for all other liquid alkane products. The D content in the residual solid polymer was measured using ATR FTIR and ^2^H NMR spectroscopy (Figures 1e and S7). The spectra show bands at 2171, 2136, and 2091 cm^–1^, corresponding to C–D stretching vibrations. Standard peaks due to C–H stretching vibrations in nondeuterated CH_2_ groups appear at 2914 and 2848 cm^–1^.^40^ Quantitative estimates reveal that the solid residue is deuterated less than the liquid products (Figure 1). Nevertheless, like liquid products, the solid residue exhibits a sharp decrease in deuterium fraction at ω­(octane) ≈ 0.21.

It is known that C–H and C–D bond formation and breaking are quasi-equilibrated under hydrogenolysis conditions, with C–C bond breaking being slower.^17^ All components of the mixture, C_8_H_18_ (H-octane), HDPE, and D_2_, actively participate in exchange reactions. Adding more H-octane to the reaction mixture at fixed amounts of D_2_ and HDPE lowers the D atom fraction and reduces the deuteration of the reaction products. Thus, deuteration decreases linearly with ω­(octane). At ω­(octane) ≥ 0.21, deuteration drops sharply as the catalyst activity reaches a plateau (Figure 1b). Since the Ru/TiO_2_ surface is covered with a high fraction of octane, octane may alter the distribution of H or D on the catalyst. To probe the properties of the octane-saturated surface, we added fully deuterated *d*_18_-octane to the reaction mixture instead of standard H-octane.

The extent of deuteration was compared between D_2_ and *d*_18_-octane as D-sources under identical reaction conditions (Figure 2). The total atomic fraction of deuterium D/(H + D) was kept constant to ensure comparable cases on an isotopomer basis (Figure S9). The octane weight fraction was also constant at ω­(octane) = 0.4. In the case of deuteration with *d*_18_-octane, liquid products incorporated a substantial number of D atoms (Figure 2a). Liquid product *n*-decane was deuterated ∼4 times more with *d*_18_-octane than with D_2_ gas. The solid residue was slightly less deuterated with *d*_18_-octane than with D_2_. Isotope analysis of the recovered octane solvent after reaction shows that octane exchanges one D or H atom (Figure S9). No olefins were detected in the product mixture.

**Figure 2:**
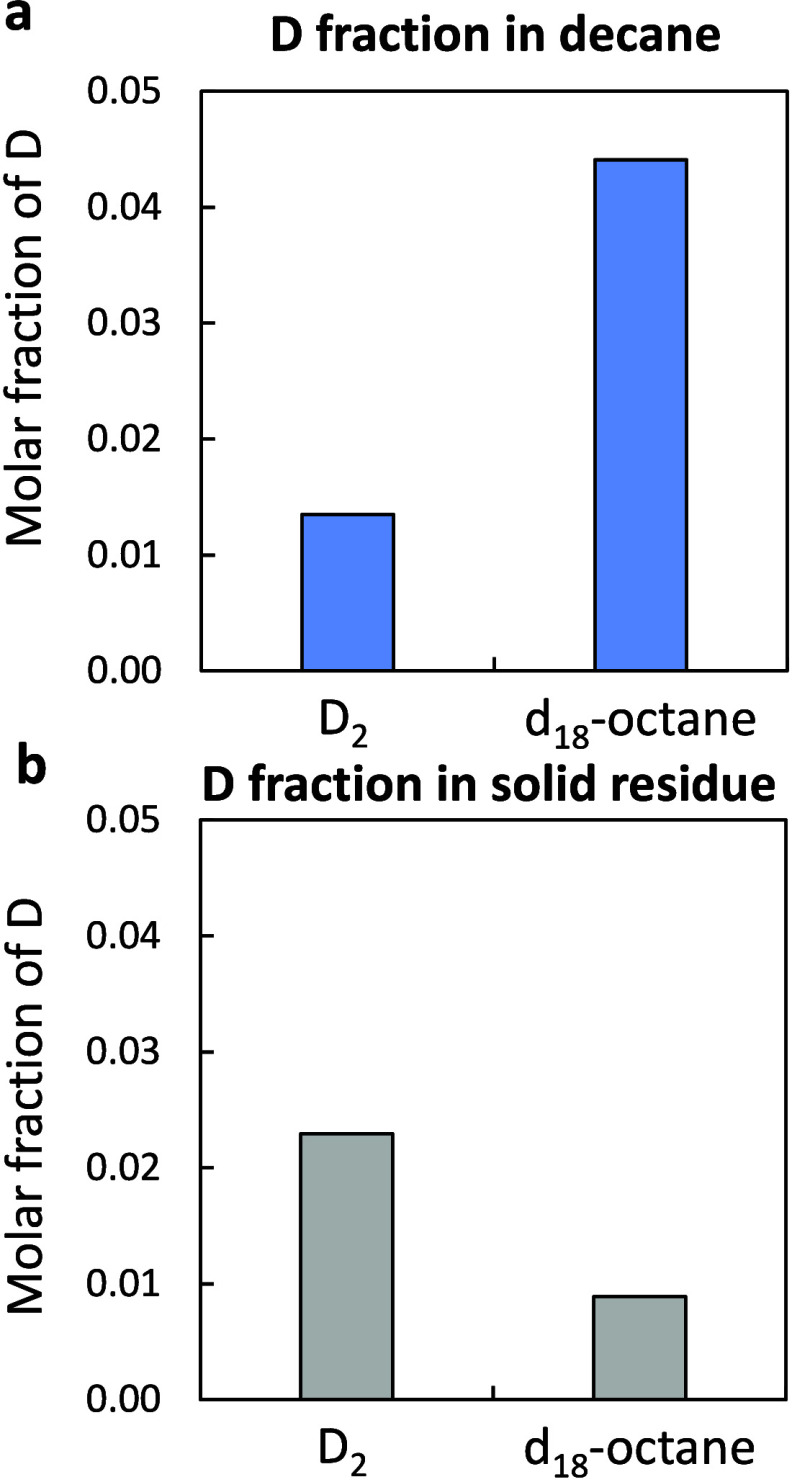
Effect of the D source on the deuteration outcome. Fraction of D atoms in (a) the liquid reaction product represented here with *n*-decane and (b) the solid residue after reaction for two reaction mixtures: (i) D_2_ gas, 2 g of HDPE, and 2.7 g of *n*-octane C_8_H_18_, and (ii) H_2_ gas, 2 g of HDPE, 1.9 g of *n*-octane (C_8_H_18_), and 0.9 g of *d*_18_-octane (C_8_D_18_). Conditions: 30 bar of H_2_ (or D_2_), 250 °C, and 10 min.

A Ru surface saturated with octane at ω­(octane) ≥ 0.21 retards D_2_ adsorption, leading to a low surface concentration of D_ads_ derived directly from gas D_2_. In this case, octane solvent serves as a hydrogen donor on the catalyst surface. The top cycle in Figure 3 shows that *n*-octane will dissociate into surface-bonded species (likely alkyls)^41^ and H_ads_. Further hydrogenation of alkyls with H_2_ will cause their desorption without the breakage of the C–C bond. H_ads_ derived from octane can participate in hydrogenolysis reactions on the Ru surface. The bottom cycle in Figure 3 shows the formation of shorter alkyl intermediates from the chemisorbed polyethylene chain. H_ads_ can saturate these intermediates, leading to their desorption as reaction products. This explains why *d*_18_-octane causes a lot of D-transfer to reaction products, like *n*-decane. Thus, both cycles in Figure 3 should operate synchronously in octane-mediated hydrogenolysis. Quantitative estimates based on Figure S9 show that *n*-octane undergoes C–H or C–D bond cleavage at $r_{H-D}=10.1\frac{{\mathrm{mmole}}_{C-\mathrm{H bonds}}}{L\cdot s}$ , which corresponds to the upper cycle in Figure 3. Data on the C–C bond breaking (Table S1) indicates that it occurs at $0.3\frac{{\mathrm{mmole}}_{C-\mathrm{C bonds}}}{L\cdot s}$ , which is 30 times slower than simple H–D exchange in octane. Thus, only 1 out of 30 H_ads_’s from the top cycle in Figure 3 participates in the bottom cycle.

**Figure 3:**
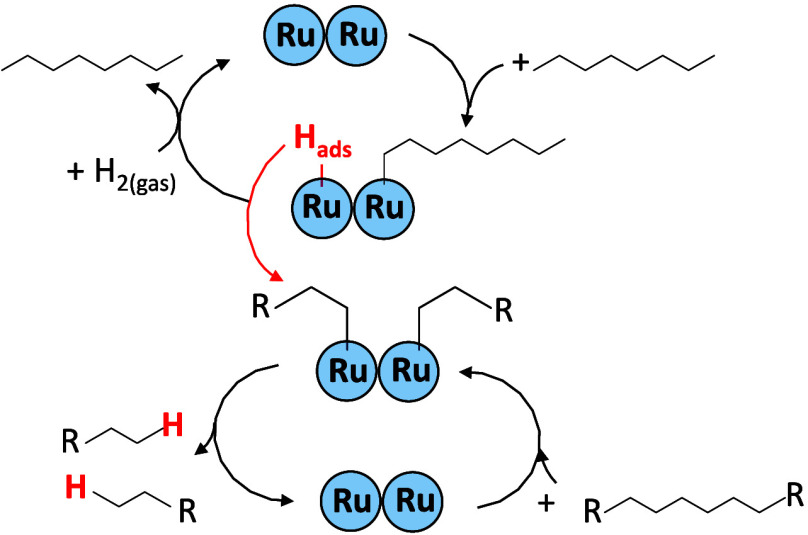
Hydrogen distribution modes on the Ru surface. Octane dissociative adsorption creates chemisorbed hydrogen, which is consumed in the hydrogenation of chemisorbed alkyl species derived from the polymer.

Direct H_ads_ exchange between chemisorbed hydrocarbons on metal surfaces without evolution or involvement of gas-phase hydrogen is well known in the cyclohexene disproportionation to cyclohexane and benzene.^42^ H_2_ release via 2H_ads_ → H_2_ is inhibited due to fast H-transfer between chemisorbed species and low θ_H_. Zhang et al.^10^ observed a similar effect in the high-temperature aromatization of polyethylene over the Pt surface, where C–C hydrogenolysis occurred under H_2_-free conditions. For the hydrogenolysis of HDPE–octane mixtures, direct H-transfer on the Ru surface may be the key to forming reaction products.

According to the reaction scheme in Figure 3, over an octane-saturated Ru surface gas-phase H_2_ reacts only with octane-derived intermediates and not with chemisorbed HDPE. This is consistent with different kinetics and metal ensemble site requirements for H–D exchange and C–C bond breaking in alkanes.^43,44^

The H–D exchange in polyolefins and octane does not occur in the melt phase; it requires a catalyst and serves as a sensitive probe of the polymer’s accessibility to the Ru surface.^45^ Octane dissociates on the Ru surface from the octane–HDPE melt and then undergoes H–D exchange. The competitive adsorption of octane explains the decrease in HDPE conversion at ω­(octane) 0.02–0.1. At ω­(octane) = 0.21, the catalyst becomes saturated with adsorbed octane, and the hydrogenolysis mechanism changes: hydrogen is transferred directly between surface alkyl intermediates, while H_2_ dissociation is limited. Based on the HDPE conversion data (Figure 1), this change in the hydrogen transfer mechanism does not preclude polymer adsorption and conversion, which remains below the octane-free case.

### 2.2 Effect of Octane Addition on Hydrogenolysis Outcome

Adding octane reduces the intrinsic HDPE conversion rate at short reaction times. Still, polymer hydrogenolysis involves a sequence of C–C bond scissions.^46^ The implications of octane addition over a 0.5–4 h period are shown in Figure 4 and Table S5.

**Figure 4:**
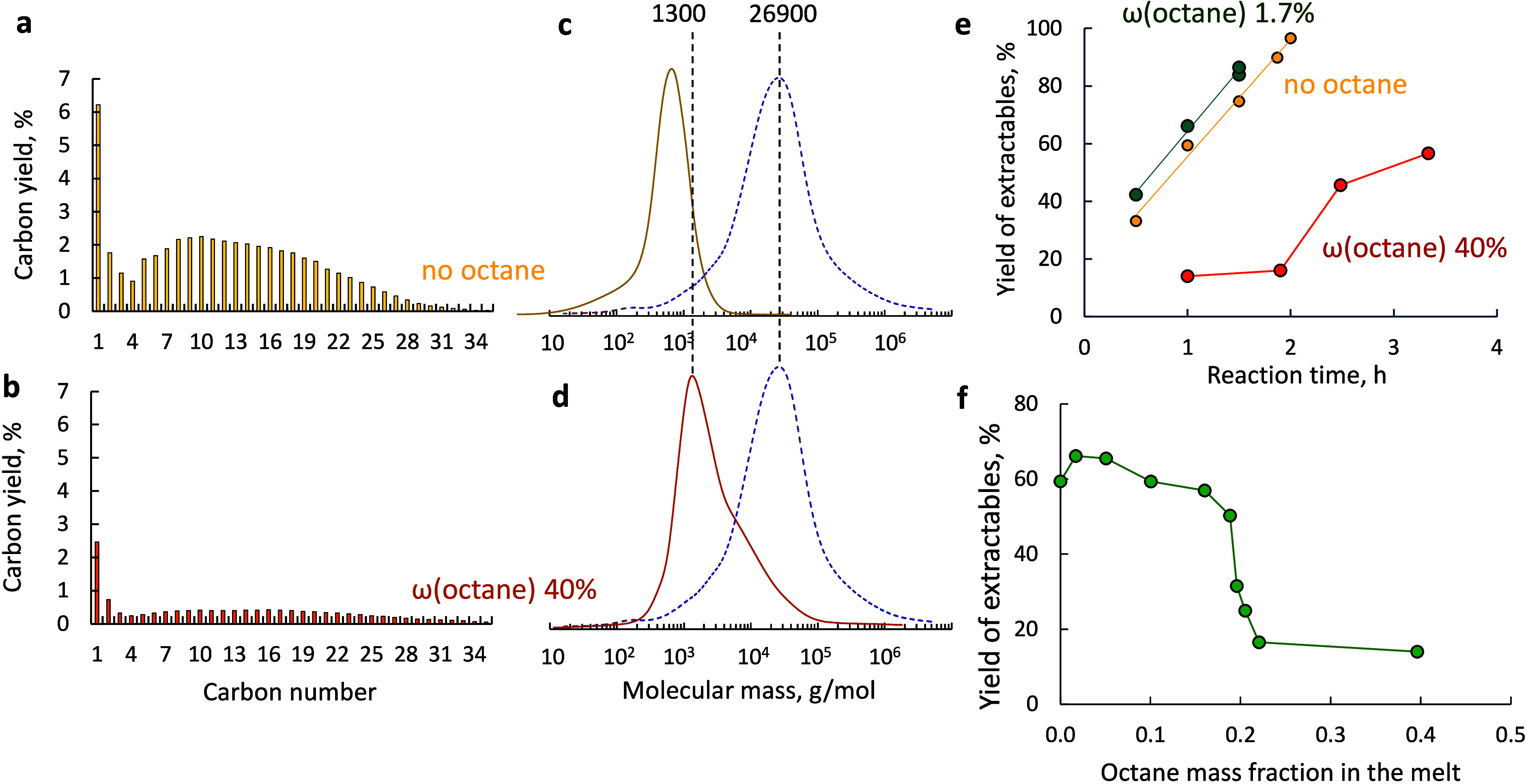
(a, b) Liquid hydrocarbon distribution for the reaction of pure HDPE and an HDPE–octane mixture for 1 h. (c, d) Corresponding molecular weight distributions for solid residues (dashed lines represent pristine HDPE). (e) Yield of extractables as a function of reaction time for different HDPE–octane mixtures. (f) Yield of extractables after 1 h of reaction as a function of the *n*-octane mass fraction in the melt. Conditions: 250 °C, 30 bar H_2_, 2 g of HDPE, 50 mg of Ru/TiO_2_ catalyst, and *n*-octane addition in the 0–2.7 g range.

The typical product distribution in Ru-catalyzed hydrogenolysis^21,40^ entails a high yield of methane and a diverse set of C_5_–C_20_ linear alkanes (Figure 4a,b). GPC analysis showed complete conversion of pure HDPE within 1 h, as evidenced by the disappearance of the peak corresponding to the pristine polymer. The remaining solid residue consists primarily of C_30_–C_90_ alkanes, which have low solubility in the dichloromethane washing solvent (Figure 4c,d). The yield of the extractables increases linearly with time, reaching 97% in 2 h (Figure 4e). The liquid product composition does not change, consistent with a previous report.^22^

The addition of a small fraction of *n*-octane [ω­(octane) = 0.02–0.1] increases the extractable yields by 11–15% at all reaction times (Figure 4e). The constant slope of the yield–time curve indicates that the *n*-octane addition affects only the early stages of the reaction. Increasing the *n*-octane content to ω­(octane) = 0.4 results in a sudden drop in extractable yield, characterized by a sharp transition at ω­(octane) ≈ 0.2 (Figure 4f), a 4-fold decline in extractable C_1_–C_35_ alkanes, and a 2.5-fold decrease in methane yield. The solid residue becomes heavier, and the maximum in the molecular weight distribution shifts from 700 to 1300 g/mol (Figure 4c,d). The molecular weight distribution contains several overlapping peaks, and the *M*_n_ increases from 600 to 3100 g/mol. At ω­(octane) = 0.40, the yield of extractables exhibits an induction period of 2–2.5 h, followed by a slow increase and leveling off at ∼60%. It indicates substantial inhibition of C–C bond scission by *n*-octane in the early and late stages of the reaction. Therefore, both the polymer conversion and liquid production are lower.

The sharp decline in extractable yield at ω­(octane) ≈ 0.2 (Figure 4f) coincides with the formation of an *n*-octane-saturated surface. While the initial HDPE reactivity is only 30% slower over the *n*-octane-saturated Ru surface, sequential C–C bond breaking is impeded more. In excess octane, HDPE converts to C_30_–C_90_ alkanes, which remain in the melt and are further slowly cracked into extractable products with an induction period of ∼2–2.5 h (Figure 4e).

Adding small amounts (2–10%) of *n*-octane decreases polymer reactivity due to competitive binding on the Ru surface, but it does not significantly impact liquid yields at longer reaction times. Thus, small amounts of *n*-octane impede the initial conversion of HDPE but have less impact on the hydrogenolysis of short- and medium-sized alkanes.

At ω­(octane) > 0.2, *n*-octane saturates the surface and inhibits the hydrogenolysis of intermediate heavy alkanes, leading to an induction period on the yield–time curve. Likely, *n*-octane suppresses alkane and H_2_ chemisorption, decreasing the hydrogenolysis rate. To rule out potential contamination of the catalyst with minor impurities from *n*-octane, we treated Ru/TiO_2_ with octane for 2 h, followed by an *n*-octane-free HDPE hydrogenolysis reaction (Figure S10). These reference experiments demonstrated that *n*-octane or any impurities in it do not deactivate the catalyst, and the inhibition is solely caused by *n*-octane adsorption from the melt. In the solvent-free hydrogenolysis of polyolefins, reaction products serve as solvents, potentially leading to a similar inhibition.

Octane was not completely inert under the reaction conditions and exhibited up to 10% conversion due to hydrogenolysis after 1 h (Table S3). During this reaction time, ∼10^3^ μmol of octane undergoes hydrogenolysis, while ∼71 × 10^3^ μmol of C–C bonds from HDPE is broken. This semiquantitative rate comparison indicates that C–C bond scission in octane is an order of magnitude smaller relative to that in HDPE and other heavier alkanes.

Octane also alters the product selectivity. The extractable fraction distributions for various octane loadings are shown in Figure 5a. In the *n*-octane-free hydrogenolysis, the catalyst produces more methane and a smaller quantity of C_20–30_ alkanes. The addition of octane allows shifting the distribution toward heavier products: the C_1–3_ fraction is reduced from 0.19 to 0.10. The fraction of heavier C_>20_ alkanes for the *n*-octane-free reaction increases from 0.37 to 0.57. The “middle portion” of the liquid distribution (C_15–19_) is unaffected (Figure S10). The effect is similar for ω­(octane) from 0.02 to 0.40.

**Figure 5:**
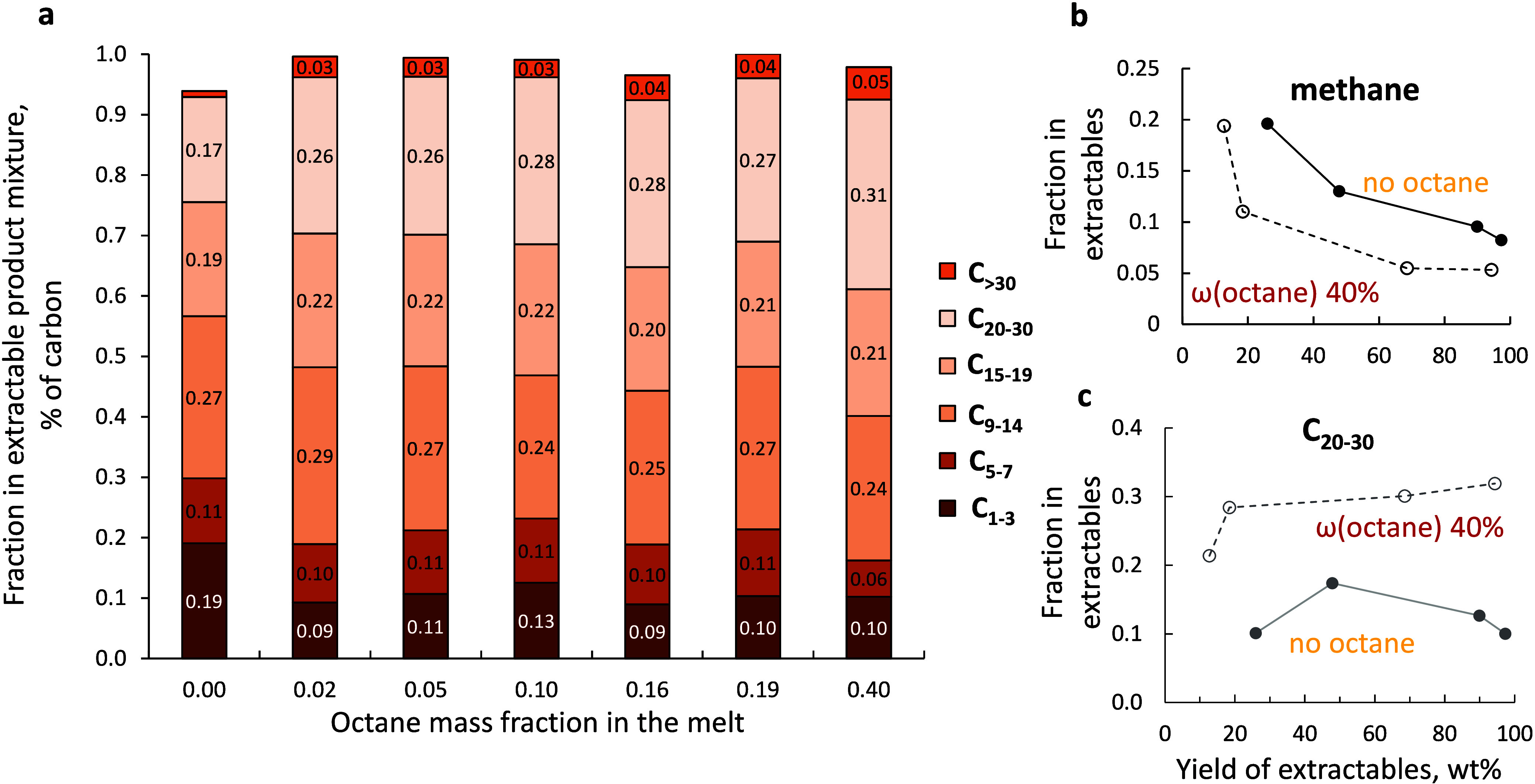
(a) Fraction of product groups by carbon number vs octane mass fraction at ∼60% yield of extractables. (b, c) Fractions of methane and C_20–30_ alkanes vs the total yield of extractables for the pure HDPE reaction and a mixture of HDPE and 40% octane. Conditions: 250 °C, 30 bar of H_2_, 2 g of HDPE, and 50 mg of Ru/TiO_2_ catalyst.

This shift in product distribution is illustrated by plotting fractions of methane and C_20–30_ alkanes as a function of the yield of extractables (Figure 5b). In the early stages of the reaction (low yield of extractables), HDPE primarily reacts to form a solid residue and methane, leading to a relatively high methane fraction, which decreases later. The addition of *n*-octane solvent allows us to reduce the methane fraction by 1.5- to 2-fold, especially at early stages (Figures 5b and S11). The reverse effect is evident for heavier products, where the yield of C_20–30_ increases by 2.5-fold.

A similar change in the product distribution can be accomplished using a high pressure of H_2_ or higher *θ*_H_, where hydrogen facilitates the desorption of medium-chain-length intermediates.^23^ Since octane saturation of the catalyst surface achieves a similar effect, we speculate that an octane-saturated surface facilitates reaction intermediate desorption off the catalyst surface before secondary terminal scission happens.

### 2.3 Effect of Solvent *n*-Alkane Chain Length and Structure

Octane, *n*-hexadecane (C_16_H_34_), and *n*-tetracosane (C_24_H_50_) were used to assess the effect of the solvent chain length (Figure 6a,b). Longer alkanes substantially decrease the extractable yield, even at a mass fraction of only 0.05 (Table S4). The molar mass distribution of solid residue indicates that tetracosane leaves ∼5% of residual polymer unreacted after 1 h, compared to no polymer left in a solvent-free experiment (Figure 6b). At the same time, the reactivity of the added solvent increases with increasing chain length. Tetracosane exhibits nearly complete conversion, whereas *n*-octane shows only minimal reactivity. This trend in small alkane conversion correlates with general observations on the higher hydrogenolysis reactivity of longer alkanes.^47^

**Figure 6:**
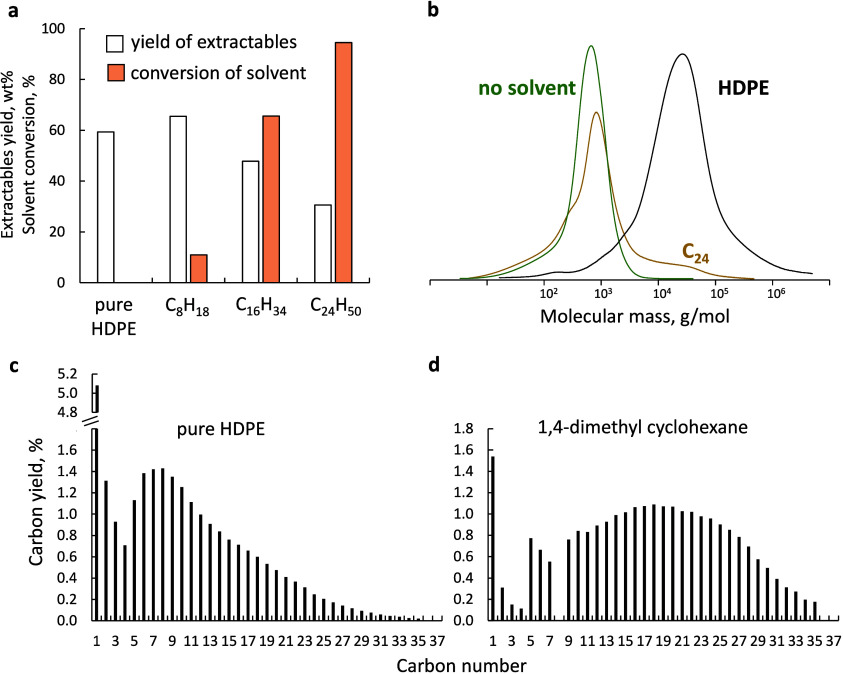
(a) Yield of extractables and solvent conversion for different linear alkanes after 1 h of reaction. (b) Molar mass distribution of solid residues after reaction without solvent and with tetracosane. (c, d) Product distribution by carbon number for the reaction of 2 g of pure HDPE for 0.5 h and a mixture of 2 g of HDPE and 2.6 g of 1,4-dimethyl cyclohexane for 2 h.

Cycloalkanes can also substantially alter the activity and selectivity of the catalyst (Figure 6c,d). Like *n*-octane, adding 1,4-dimethylcyclohexane results in low extractable yields (Table 1). The products are heavier (C_20_–C_30_) than pure HDPE and HDPE-*n*-octane mixtures, and the methane yield decreases to 1.5%, which is 3.4 times lower than that of pure HDPE. Decalin, another cyclic compound, almost completely inhibits catalyst activity, with minimal extractable yields after 1 h with the formation of some decalin ring-opening products. Decalin likely undergoes partial dehydrogenation and forms surface-bonded species, which serve as hydrogenolysis inhibitors, which explains a suppressed H–D exchange in polyolefins at 170 °C over the Pt–Re/SiO_2_ catalyst.^31^ Recent work on converting plastics containing additives demonstrated that aromatic rings bind too strongly to metals to desorb at typical hydrogenolysis reaction temperatures.^48^

**Table 1:** Effect of the Added Cycloalkane Structure on HDPE Hydrogenolysis^a^

				Fraction of extractable product mixture	
Solvent	Amount added, g	Reaction time, h	Yield of extractables, %	methane	C_20–30_
-	-	2	97.3	0.08	0.10
*n*-octane	2.7	1.9	11.3	0.13	0.28
1,4-dimethyl-cyclohexane	2.6	2	16.2	0.06	0.37
decalin	2.6	2.3	0.7	-	-
decalin	0.1	1	3.4	0.22	0.11
-^b^	-	0.5	25.9	0.20	0.10

a Conditions: 250 °C, 30 bar H_2_, 2 g of HDPE, and 50 mg of Ru/TiO_2_ catalyst.

b Comparative data at an approximately similar yield of extractables.

DFT calculations were employed to determine the binding strength of various alkanes and cycloalkanes to Ru and correlate it with catalytic performance (Table 2 and Figure S12). The binding energies for *n*-octane and 1,4-dimethyl cyclohexane are relatively similar, indicating that the difference in methane suppression does not originate from simple binding to the surface. Due to its six-membered ring, 1,4-dimethylcyclohexane can block larger patches of the Ru surface. Its deep dehydrogenation into a benzene derivative can supply hydrogen atoms for hydrogenolysis and reduce methane selectivity, but it also suppresses reactivity.

**Table 2:** DFT-Derived Energies and Equilibrium Adsorption Constants for Alkane Physisorption onto Ru(0001)

	Octane	1,4-Dimethyl-cyclohexane	Decalin	Hexadecane
Δ*E*, kJ/mol (ZPE-corrected), 0 K	–113.9	–104.2	–125.4	–210.4
Δ*G* ^0^_250 °C_, kJ/mol	–17.4	–15.4	–28.0	–75.6
$K_{eq}=e^{-\Delta G_{250C}^{0}/RT}$ at 250 °C	59.7	35.1	614.8	3.6 × 10^7^

Modeling shows that *n*-hexadecane has a 2.8 times higher binding energy than decalin when normalized to the number of carbon atoms. Experiments indicate that the hydrogenolysis of HDPE is suppressed by hexadecane (Figure 6a) but not as severely as by decalin. It shows that binding energy is not a good descriptor for explaining the poisoning effect of cycloalkanes over *n*-alkanes. More likely, decalin partially dehydrogenates into surface-bound aromatic compounds, which bind to the surface and alter metal electronic properties.^49^ On the other hand, the binding energy is a good descriptor of the effect of *n*-alkane chain length on hydrogenolysis. Longer-chain *n*-alkanes bind more strongly, inhibiting more.

### 2.4 Effect of Polymer Type and Molecular Weight

Low-molecular-weight LDPE (*M*_n_ ≈ 2.2 kg/mol) and a mixture with *n*-octane were tested to estimate the impact of the chain length of the initial polymer (Figure 7a). The yield of extractables increases more slowly for pure LDPE than for HDPE due to slower hydrogenolysis of more branched polyethylene. The liquid from LDPE forms more slowly at ω­(octane) = 0.4 than in the solvent-free case. Octane-induced inhibition is less pronounced, resulting in a decline in extractable yield from 76 to 63 wt % over 2 h. At longer reaction times, the extractable yield flattens at ∼70 wt %, similar to that of the HDPE-*n*-octane mixture (Figure 4e).

**Figure 7:**
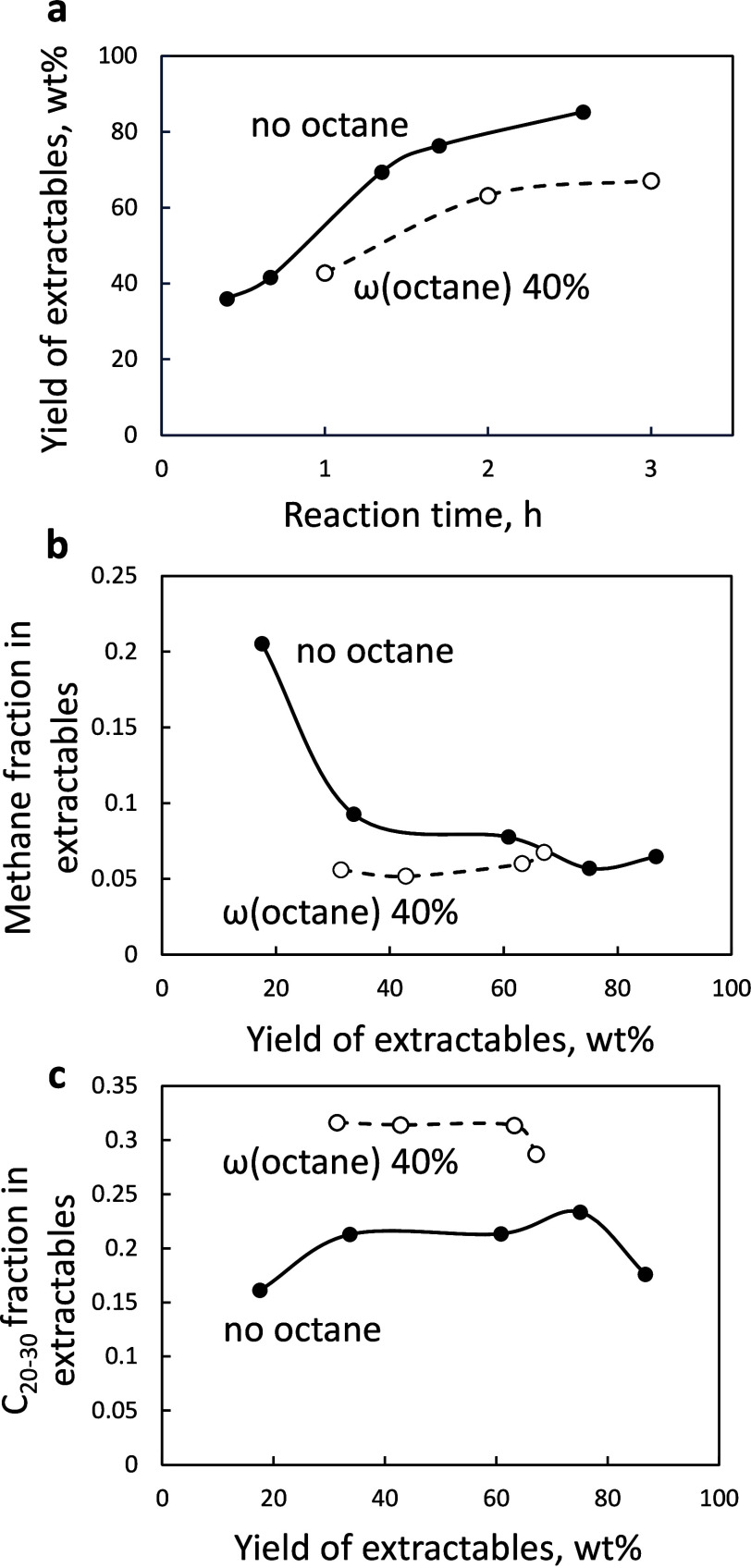
(a) Yield of extractables vs reaction time for LDPE-*n*-octane mixtures. (b, c) Fractions of methane and C_20–30_ alkanes vs total yield of extractables for pure LDPE reaction and a mixture of LDPE and 40 wt % *n*-octane. Conditions: 250 °C, 30 bar H_2_, 2 g of LDPE, and 50 mg of Ru/TiO_2_ catalyst.

Octane reduces the methane fraction in the product mixture (Figure 7b) in the early stages of the reaction. The addition of *n*-octane increases the C_20_–C_30_ fraction by 1.5 times, consistent with the HDPE results.

Over Ru/TiO_2_ catalysts, polypropylene (PP) is converted into a lubricant-range oil with a small amount of methane byproduct.^13^ Figure 8 shows the results of the PP-*n*-octane mixture hydrogenolysis to liquids. The addition of *n*-octane increased the solid yield. The liquid yield reaches a maximum of 70 wt % at 12 h instead of 6 h in the *n*-octane-free case (Figure 8b). The liquid molecular weight was consistently higher for the PP-*n*-octane mixture. It displayed a very subtle decrease due to the inhibition of C–C bond scission in the late reaction stages. We observe the same flattening at high reaction times over *n*-octane-saturated surfaces for all polymers studied in this work.

**Figure 8:**
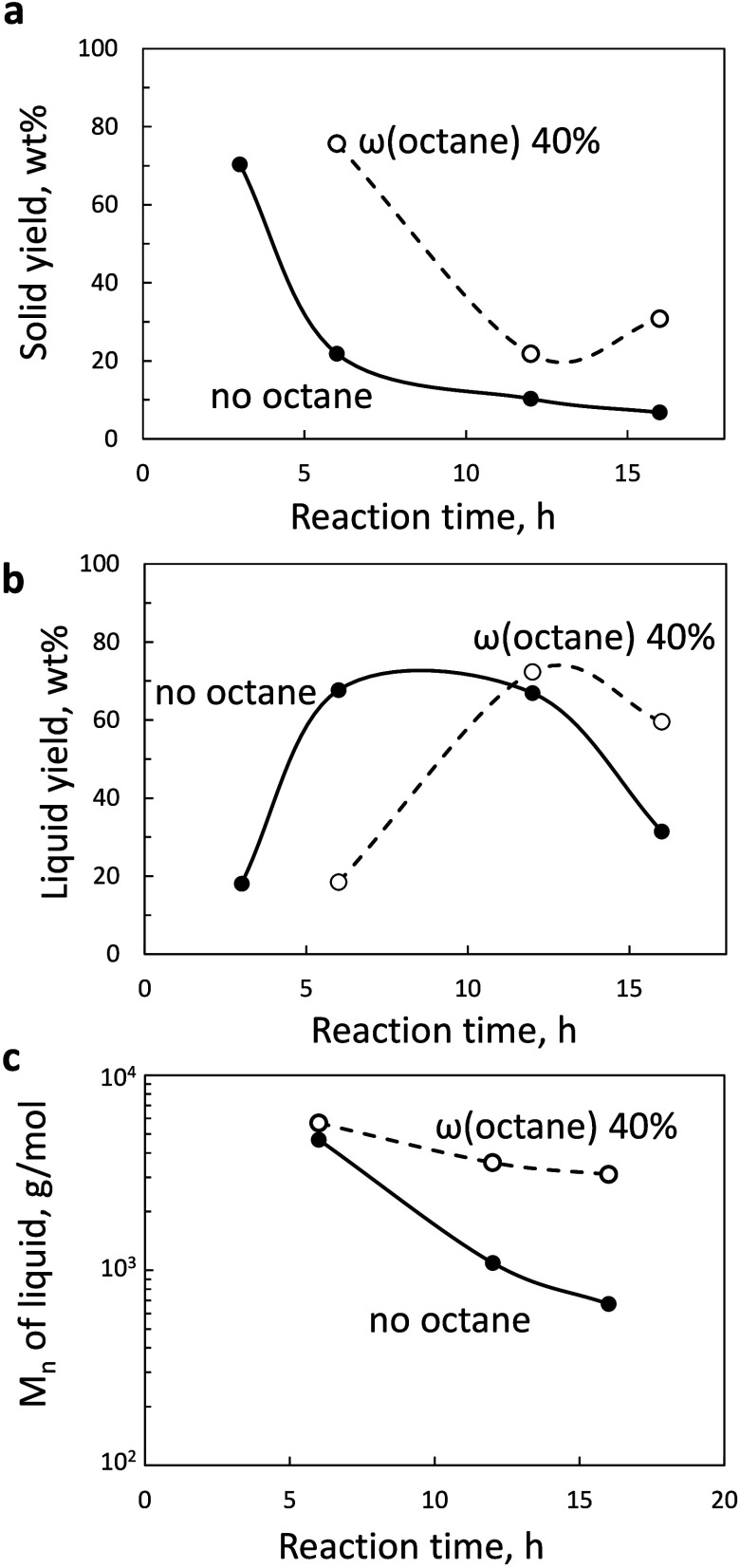
(a) Solid residue yield and (b) liquid yield as a function of reaction time for pure PP and PP mixed with *n*-octane. (c) *M*_n_ of liquid products of PP hydrogenolysis. Conditions: 250 °C, 30 bar H_2_, 2 g of PP, and 50 mg of Ru/TiO_2_ catalyst.

## 3 Conclusions

This work investigated the impact of alkanes and cycloalkanes as solvents on the reactivity of POs over Ru/TiO_2_, which is a common catalyst for hydrogenolysis. Such compounds form in high concentrations at high conversions during many deconstruction processes. Our original hypothesis was that solvents can reduce viscosity, increase diffusion, enhance the mixing of catalyst and reactants, and improve reactivity. Polymer conversion data indicate that the impact of an alkane solvent depends on its concentration and the stage of the process. For example, to our surprise, *n*-octane, used as a prototype solvent, reduces the hydrogenolysis rate of the initial HDPE polymer due to competitive adsorption and impacts the sequential C–C bond breaking in intermediate C_30_–C_90_ reaction products less. Longer *n*-alkanes inhibit hydrogenolysis more, due to the higher binding strength of *n*-alkanes as solvents on Ru. This effect also occurs for LDPE and HDPE.

The reduction in reaction rate is significant at *n*-octane weight fractions in the melt above ∼0.2 due to the formation of an *n*-octane-saturated surface. In this case, hydrogenolysis shows an induction period and a plateau at intermediate conversion levels. Isotope analysis using D_2_ and *d*_18_-octane showed that an *n*-octane-saturated surface allows direct H-transfer from *n*-octane to the chemisorbed reaction intermediate. The dissociation of gas-phase H_2_ is hindered due to high *n*-octane surface coverage. Interestingly, the *n*-octane-saturated surface is initially active but becomes significantly less active in subsequent sequential C–C bond breakings. Intermediate-size alkanes compete with excess *n*-octane for access to the catalyst surface.

Octane and 1,4-dimethyl-cyclohexane reduce the methane fraction and increase the C_20_–C_30_ part of the product distribution. Cyclic solvents retard the rate even more, probably due to dehydrogenation and the formation of strongly binding aromatics.

Important implications arise from the inhibiting action of alkanes. During solvent-free polymer hydrogenolysis, the reaction products may act as self-inhibitors of the reaction. The threshold for this effect is ∼5 wt % for longer alkanes such as hexadecane. A substantial acceleration of the reaction rate would be expected if the reaction products were continuously removed from the reaction mixture. Future research directions will involve improvements in hydrogenolysis productivity with remaining high selectivity for desirable liquid products.

## 4 Methods

### 4.1 Catalyst Preparation

The Ru/TiO_2_ catalysts were prepared by wetness impregnation using a commercially available anatase TiO_2_ support (U.S. Research Nanomaterials). Before impregnation, the TiO_2_ powder was calcined at 450 °C for 6 h in static air. Ru/TiO_2_ was prepared by mixing 2.77 g of Ru precursor solution (ruthenium­(III) nitrosyl nitrate solution in diluted nitric acid, Sigma-Aldrich) with 1 g of deionized water and several drops of aqueous NH_3_ (25%, Supelco) to increase the pH to 8. The solution was added to TiO_2_ powder under manual stirring with a glass rod at 70 °C. After impregnation, the catalyst was dried at 100 °C overnight and reduced in H_2_ (50 vol % in He) flow in a tubular furnace at 300 °C for 2 h (ramp rate 10 °C/min).

### 4.2 Reaction Tests

High-density polyethylene (HDPE, *M*_n_ ≈ 6 kg/mol, weight-averaged molecular weight (*M*_w_) ≈ 122 kg/mol), isotactic polypropylene (PP, *M*_*w*_ ≈ 350 kg/mol), and low-density polyethylene (LDPE, *M*_w_ ≈ 2.2 kg/mol) were purchased from Sigma-Aldrich (catalog numbers 427985, 427861, and 428043, respectively). Freshly reduced catalyst (0.1 or 0.05 g) was mechanically mixed with 2.0 g of polymer granules by using a vortex mixer. The mixture was then transferred into the borosilicate liner of a 50 mL stainless-steel Parr reactor with a 0.7 mL stir bar. Then, the required amount of liquid alkane solvent was added using a calibrated pipet. The Parr reactor was sealed and purged six times with pure H_2_ at 10 bar, charged to 30 bar, and then heated to 250 °C (ramping rate 15 °C/min) using a 300 W band heater (Tempco) connected to a PID controller. Stirring was initiated at 500 rpm after the temperature reached 160 ± 5 °C, so the polymer had melted. Reactions were maintained for specified time intervals and then quickly quenched in an ice bath.

In experiments with isotope labeling, the reactor was charged with D_2_ (Sigma-Aldrich 617474) instead of H_2_, or fully deuterated octane (Sigma-Aldrich 151971) was used in a mixture with regular *n*-octane.

### 4.3 Product Analysis

After the reactor temperature dropped below 10 °C, the gas from the reactor’s headspace was transferred to a 1 L Tedlar gas sampling bag for analysis. Then the reactor was opened, and the liquid and solid residue were mixed with 20 mL of CH_2_Cl_2_ (containing 20 mg of *n*-octacosane as an internal standard). This slurry was filtered (Whatman, 100 μm), and the solid residue was dried at room temperature overnight with complete evaporation of all CH_2_Cl_2_. The solid fraction yield was quantified gravimetrically. Liquid products were analyzed using gas chromatography with a flame ionization detector (GC-FID) with an HP-5 column. Calibration coefficients and retention times for all products were measured using C_1_–C_35_ analytical standards.

A GC-FID instrument (Agilent 7890 series, HP-volamine column) was used for gas analysis. The concentration of hydrocarbons in the gas sample was calculated using a standard C_1_–C_4_ calibration mixture. The absolute amount of hydrocarbons in the gas was calculated by using the ideal gas law. An overall balance was calculated as follows

$$materialbalance=\frac{m_{L}+m_{s}+m_{g}}{m_{initial}}\times 100wt\%$$

wherein *m*_L_, *m*_s_, *m*_g_, and *m*_initial_ are the mass of liquid, gas, solid, and initial polymer, respectively. Liquid products from polypropylene conversion were separated from CH_2_Cl_2_ solvent using a rotary evaporator and analyzed gravimetrically and with gel permeation chromatography (GPC) using Styragel HR 4, HR 3, and HR 0.5 columns (dimensions 4.6 × 300 mm^2^) connected in tandem using THF as a solvent (0.3 mL/min flow rate) and a Waters 2414 refractive index detector (RID). The retention time was calibrated using a polystyrene standards kit (Waters, WAT058931).

The molar carbon yield of the product group with *i* carbon atoms was calculated as

$$Y_{i}=\frac{N_{C}^{i}}{N_{C}^{HDPE}}$$

where *N*_C_^*i*^ is the number of moles of carbon in the product and *N*_D_^HDPE^ is the number of moles of carbon in the initial polymer. The fraction of the *i*th product group was calculated as

$$F_{i}=\frac{Y_{i}}{\underset{j}{\sum }Y_{j}}$$

where ∑_*j*_ *Y*_*j*_ is the sum among all products formed. The yield of extractables was calculated as ∑_1_^35^ *Y*_*i*_.

The rate of HDPE consumption (*r*_p_) was calculated as $\frac{CC_{initial}-CC_{t}}{V\Delta t}$ wherein *CC*_initial_ is the initial number of carbon–carbon bonds in HDPE, mol; *CC*_*t*_ is the number of carbon–carbon bonds in the solid residue at time *t*, mol; Δ*t* is the time interval corresponding to 10 min; and *V* is the volume of the reaction media, including melted HDPE and octane. The HDPE conversion was estimated as $\frac{CC_{initial}-CC_{t}}{CC_{initial}}$

Fourier transform infrared attenuated total reflectance (FTIR-ATR) spectra of the solid residue and the initial polymers were recorded by using a Nicolet Nexus 640 spectrometer with a Smart Orbit diamond ATR accessory in the 4000–650 cm^–1^ range.

### 4.4 Solid Residue Analysis Using High-Temperature GPC (HT-GPC)

GPC was performed on a high-temperature system (Tosoh HLC-8312GPC/HT with two TSKgel GMHHR-H(20)­HT columns and one TSKgel G2000HHR (20)­HT column in series) using a refractive index (RI) detector. Measurements were performed at 140 °C at a 0.8 mL/min flow of the mobile phase of 1,2,4-trichlorobenzene with 500 ppm added butylated hydroxytoluene to prevent oxidation. Injections of 300 μL of each sample were eluted for 80 min. A series of nine narrow standards of polystyrene were used to calibrate the system for 2.5 < log *M* < 6.5, and Mark–Houwink constants were used to account for the polyethylene samples measured for this work. The total concentration of all molecular weight species was measured and converted to the yield of each molecular weight solid. Details of the GPC analysis used for this work have been reported in our previous publications.^32^

Polymer conversion was calculated from the molecular weight distribution using a previously developed approach.^32^ The total number of C–C bonds in HDPE (*N*_CC_^HDPE^) and in the solid reaction products (*N*_CC_^product^) was calculated from GPC curves, and the C–C bond-breaking rate (*r*_p_) was computed as

$$r_{p}=\frac{1}{V_{r}}\frac{N_{CC}^{HDPE}-N_{CC}^{product}}{10\min}$$

where *V*_r_ is the volume of the octane–HDPE mixture.

### 4.5 SAFT-γ Mie Calculations

The dissolved hydrogen concentration in HDPE + *n*-octane mixtures was predicted using the SAFT-γ Mie equation of state (EoS)^51–53^ in combination with the Helmholtz free energy Lagrangian dual (HELD) algorithm.^54^ The SAFT-γ Mie EoS employs a group contribution approach to model fluid phase behavior and thermodynamic properties, utilizing Mie interaction potentials to represent intermolecular forces with variable attractive and repulsive ranges. HDPE and *n*-octane are modeled as linear hydrocarbons incorporating CH_2_ and CH_3_ groups. HDPE was simplified as an ∼54 kg/mol monodisperse polymer. Calculations were performed at 50 bar and 250 °C.

### 4.6 Molecular Dynamics Simulations

MD simulations were conducted using the GROMACS 2021.5 package^55^ and the GROMOS 54A7 force field.^56,57^ HDPE was modeled as a linear chain of 208 united atoms, and *n*-octane was modeled as a linear chain of 8 united atoms. The initial configurations were generated by randomly replacing atoms in a cubic cell of 40 nm. After an initial energy minimization, the system was condensed at 523 K and 500 bar in an isothermal–isobaric (NPT) ensemble for 500 ps. The last configuration was used as a starting point for a 10 ns NPT equilibration at 50 bar and 250 °C. Next, the density was corrected to match the predictions obtained from the SAFT-γ Mie EoS, and a 200 ns run was performed using the canonical ensemble. The diffusion coefficients were calculated from the last 100 ns of the trajectory using the Einstein–Smoluchowski method.^58^

All simulations were performed using the Verlet algorithm^59^ and a time step of 1 fs with a cutoff distance for Lennard-Jones interactions set to 1.4 nm. The Nosé–Hoover thermostat^60,61^ was fixed with a damping parameter of 100 fs. The pressure was controlled using the Parrinello–Rahman barostat.^62^

### 4.7 Density Functional Theory (DFT) Calculations

DFT calculations were carried out using the Vienna ab initio simulation package (VASP 5.4.1).^63,64^ A frozen-core, all-electron projector augmented-wave (PAW)^65^ method was used to avoid the singularities of Kohn–Sham wave functions at nuclear positions. We treated the p states of Ru metal atoms as valence electrons such that 14 electrons in total were considered to be valence electrons for Ru. The exchange-correlation functional was expressed using the revised Perdew–Burke–Ernzerhof (RPBE) functional, developed by Hammer et al.^66^ Semiempirical dispersion corrections based on Grimme’s DFT-D3 ^67^ method were also utilized. Brillouin zone integrations have been performed with a 4 × 4 × 1 Monkhorst–Pack^68^ k-point grid for *n*-octane, 1,4-dimethylcyclohexane, and decalin, and a 2 × 4 × 1 k-point grid was used for hexadecane. Electronic wave functions at each k point were expanded using a discrete plane-wave basis set with an energy cutoff of 450 eV. A periodic slab (5 × 5 × 4) of 100 Ru was used for *n*-octane, 1,4-dimethylcyclohexane, and decalin adsorption, while a larger slab (5 × 10 × 4) of 200 Ru atoms was used for hexadecane calculations. A first-order smearing method (Methfessel–Paxton)^69^ with a 0.10 eV smearing width was employed, allowing the accurate calculation of entropic contributions due to the smearing. A self-consistent field (SCF) convergence criterion for the electronic degrees of freedom of the valence electrons was set to 1.0 × 10^–7^ eV. Structures were considered relaxed when the maximum force on any atom was less than 0.02 eV/Å. Harris corrections based on the Harris–Foulkes formalism^70,71^ have been applied to the forces and stress tensors, and the total energy was corrected for dipole effects using a modified version of the Makov-Payne scheme.^72^ The top two layers of the Ru(0001) slab were allowed to relax during geometry optimization, while the bottom two layers were fixed to their bulk positions. A 25 Å vacuum gap was employed on top of the surface, which restricted the interaction between the periodic images along the surface normal.

## Supplementary Material



## Notes

Experimental runs, GPC traces, hydrogen solubility, physicochemical framework for octane–polymer chemisorption, polymer self-diffusion coefficients, ATR spectra of a deuterated polymer, isotopomer distributions, liquid alkane composition, selectivity comparison at different conversion levels, and DFT results (PDF)
