# Supplementary material for: Impact of Small-Alkane Solvents on Polyolefin Hydrogenolysis over a Ruthenium Catalyst
Source: Ind Eng Chem Res. 2026 Apr 30;65(18):9410–22. doi: 10.1021/acs.iecr.6c00853 (PMC13178079; doi:10.1021/acs.iecr.6c00853)
Supplement: Supplementary file 1 [file ie6c00853_si_001.pdf]

Supporting information for:

Impact of small-alkane solvents on polyolefin hydrogenolysis over ruthenium catalyst

Pavel A. Kots,[1,2*] Zachary R. Hinton,[1] Mehdi Zare,[1] Brandon C. Vance,[1,3] María Ley-Flores,[4] Juan J. de Pablo,[2,4,5] Thomas H. Epps, III,[1,3,6] LaShanda T. J. Korley,[1,3,6] Michele Valsecchi[7], George Jackson,[7] Amparo Galindo,[7] Dionisios G. Vlachos[1,3*]

Affiliations:

[1]Center for Plastic Innovation, University of Delaware, 221 Academy St., Newark, DE 19716, USA

[2]Department of Chemical and Biomolecular Engineering, Tandon School of Engineering, New York University, Brooklyn, NY 11201, USA

[3]Department of Chemical and Biomolecular Engineering, University of Delaware, 150 Academy St., Newark, DE 19716, USA

[4]Pritzker School of Molecular Engineering, The University of Chicago, Chicago, IL, USA

[5]Center for Molecular Engineering, Argonne National Laboratory, Lemont, IL, USA

[6]Department of Materials Science and Engineering and Center for Research in Soft matter and Polymers (CRiSP), University of Delaware, Newark, DE 19716, USA

[7]Department of Chemical Engineering, Sargent Centre for Process Systems Engineering, Institute for Molecular Science and Engineering, Imperial College, South Kensington Campus, London, SW7 2AZ, United Kingdom

*Corresponding authors. Email: p.kots@nyu.edu, vlachos@udel.edu

Table S1. Yield of solid residue and extractables for HDPE-*n*-octane mixtures.

Weight fraction of *n*-octane in the melt	Yield of extractables, wt%	Yield of solid residue, wt%
0	1.22	98.8
0.02	1.95	98.1
0.05	2.00	98.0
0.10	1.42	98.6
0.19	2.52	97.5
0.21	2.65	97.4
0.40	2.29	97.7

Conditions: 250 °C, 30 bar H_2, reaction time of 10 min, 2 g HDPE, 50 mg Ru/TiO_2 catalyst.

Figure S1 reports the molecular weight distribution of solid residue, showing a deconvoluted Gaussian component, corresponding to a hydrogenolysis product, distinctly different from the unreacted polymer. The figure also shows the number-averaged molecular weight (M_n) of the deconvoluted product peak.

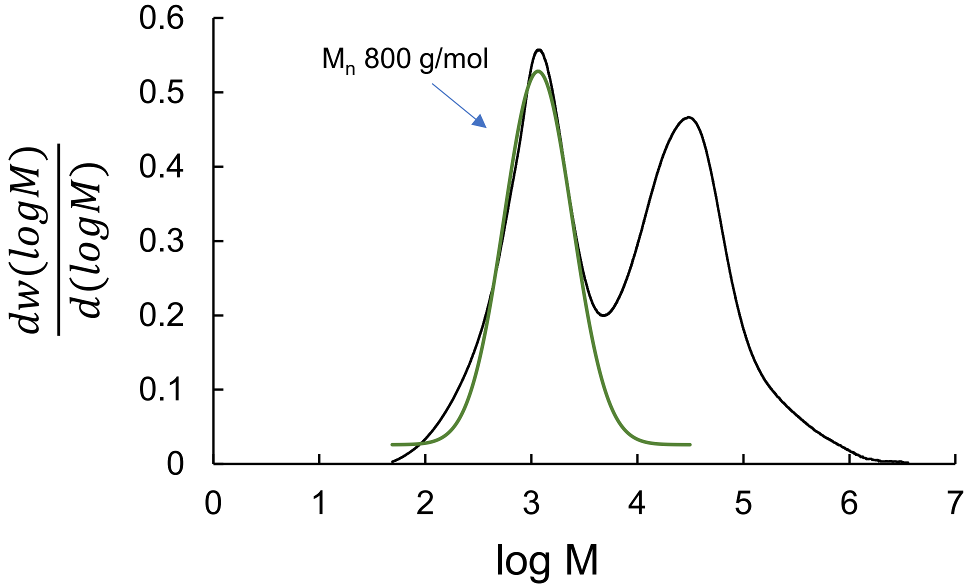

Fig. S1. Molar weight distribution for an example solid residue showing a deconvoluted peak corresponding to reaction products. Black curve – experimental data, green curve – gaussian fit for reaction product in the solid residue.[1] Conditions: 250 °C, 30 bar H_2, reaction time of 10 min, 2 g HDPE, 50 mg Ru/TiO_2 catalyst. Details of the GPC analysis are provided in the methods section.

Table S2. Effect of octane solvent on HDPE conversion.

Weight fraction of octane in the melt	HDPE conversion	M_n of the solid product, kg/mol[1]	Initial concentration of C-C bonds in HDPE, M	Reaction rate, $\frac{mmole_{C\text{-}C\ bonbds}}{L\cdot s}$
0	0.34	0.8	93.9	1.2
0.02	0.30	0.8	92.5	1.0
0.05	0.14	1.2	89.5	0.3
0.10	0.11	1.0	85.1	0.3
0.19	0.18	1.0	77.3	0.3
0.21	0.25	0.9	75.8	0.6
0.40	0.23	1.4	58.4	0.3
pristine HDPE	0.00	5.5	-	-

Conditions: 250 °C, 30 bar H_2, reaction time of 10 min, 2 g HDPE, 50 mg Ru/TiO_2 catalyst

[1] Number-average molecular weight of solid residue, $M_n = \frac{\sum \frac{dw}{dlogM}}{\sum \frac{dw}{dlogM} \cdot \frac{1}{M}}$.. [2]

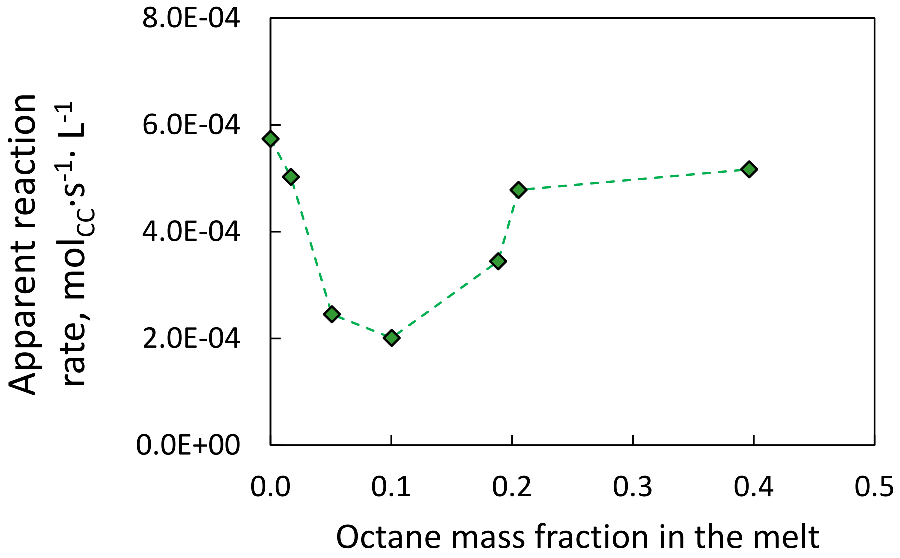

Fig. S2. Apparent reaction rate of C–C bond breaking in HDPE as a function of octane content in the melt. Conditions: 250 °C, 30 bar H_2, reaction time of 10 min, 2 g HDPE, 50 mg Ru/TiO_2 catalyst.

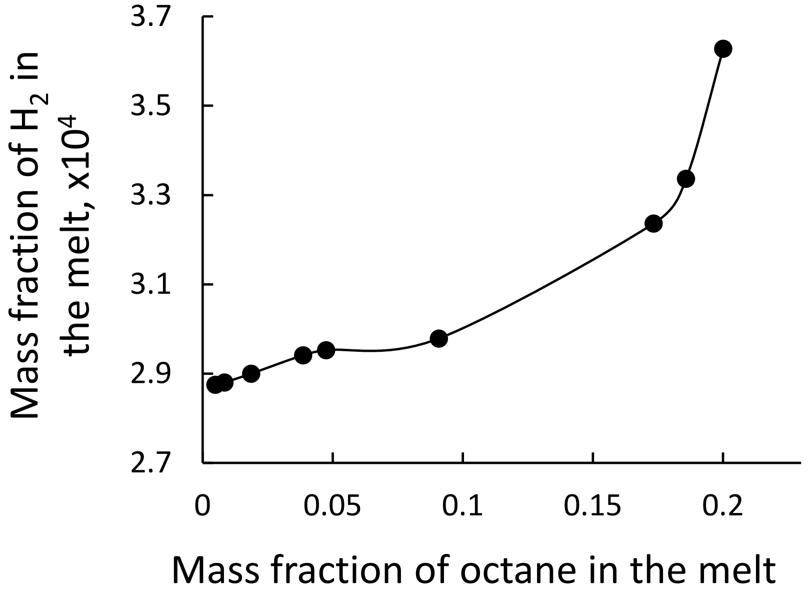

Fig. S3. Mass fraction of dissolved H_2 in HDPE/*n*-octane mixtures. Calculations made at 50 bar and 250 °C using the SAFT-γ Mie equation of state. The curve demonstrates a modest but systematic increase in dissolved H_2 up to high *n*-octane content, with a rise as the mixture approaches pure *n*-octane.

Supplementary Discussion I.

Analysis of adsorption from a polyethylene-octane solution onto the catalyst surface can be treated using the classical approach.[3] Chemical potential of octane in the chemisorbed layer on the surface is expressed using eq. S1:

$$\mu_{oct}^{a} = \mu_{oct,0}^{a} + RTln(\theta_{oct}\gamma_{oct}^{a}) \ (S1)$$

Here $\mu_{oct,0}^{a}$ is the chemical potential of chemisorbed octane in the standard state of pure octane, θ_{oct} – octane surface coverage, γ_{oct}^{a} – activity coefficient in chemisorbed layer, RT – gas constant multiplied by temperature (4.3 kJ/mol at 250 °C). Similarly, the chemical potential of octane in the liquid phase is according to eq. S2:

$$\mu_{oct} = \mu_{oct,0} + RTln(x_{oct}\gamma_{oct}) \ (S2)$$

Here x_{oct} is a molar fraction of octane in solution. If we assume thermodynamic equilibrium between chemisorbed and liquid octane, then the octane chemical potentials should be equal, yielding eq. S3 and S4:

$$\mu_{oct,0}^{a} + RTln(\theta_{oct}\gamma_{oct}^{a}) = \mu_{oct,0} + RTln(x_{oct}\gamma_{oct}) \ (S3)$$

$$\frac{\mu_{oct,0}^{a} - \mu_{oct,0}}{RT} = ln\left(\frac{x_{oct}\gamma_{oct}}{\theta_{oct}\gamma_{oct}^{a}}\right) \ (S4)$$

Similar treatment produces eq. S5 for polyethylene polymer:

$$\frac{\mu_{p,0}^{a} - \mu_{p,0}}{RT} = ln\left(\frac{x_{p}\gamma_{p}}{\theta_{p}\gamma_{p}^{a}}\right) \ (S5)$$

Here, the standard state corresponds to pure polymer melt. Dividing equations S5 and S4:

$$exp\left(\frac{[\mu_{p,0}^{a} - \mu_{p,0}] - [\mu_{oct,0}^{a} - \mu_{oct,0}]}{RT}\right) = \frac{x_{p}\gamma_{p}}{\theta_{p}\gamma_{p}^{a}} \cdot \frac{\theta_{oct}\gamma_{oct}^{a}}{x_{oct}\gamma_{oct}} \ (S6)$$

The exponent term on the left side of eq. S6 (denoted as K_s) is a thermodynamic constant that corresponds to chemisorption of pure polymer and chemisorption of pure octane. Thus, K_s is a metric of catalyst interaction with polyethylene over octane regardless of lateral interactions in solution or in the adsorbed layer. Rearranging eq. S6 gives:

$$\frac{\theta_{oct}x_{p}}{\theta_{p}x_{oct}} = \frac{\gamma_{oct}\gamma_{p}^{a}}{\gamma_{p}\gamma_{oct}^{a}}K_s(T) \ (S7)$$

Assuming adsorption from a binary solution and neglecting molar fraction of H_2:

$$x_P + x_{oct} = 1 \ (S8)$$

And surface balance, ignoring hydrogen coverage:

$$\theta_p + \theta_{oct} = 1 \ (S9)$$

Substituting eq. S8 and S9 into eq. S7 allows to express θ_p as a function of x_p:

$$\frac{\theta_{oct}x_{p}}{\theta_{p}x_{oct}} = \frac{x_p}{(1 - x_p)} \cdot \frac{(1 - \theta_P)}{\theta_p} = \frac{\gamma_{oct}\gamma_{p}^{a}}{\gamma_{p}\gamma_{oct}^{a}}K_s \ (S10)$$

$$\theta_p = \frac{x_p}{x_p\left(1 - \frac{\gamma_{oct}\gamma_{p}^{a}}{\gamma_{p}\gamma_{oct}^{a}}K_s\right) + \frac{\gamma_{oct}\gamma_{p}^{a}}{\gamma_{p}\gamma_{oct}^{a}}K_s} \ (S11)$$

Eq. S11 shows that polymer surface coverage is a function of the molecular field in solution ($\frac{\gamma_{oct}}{\gamma_p}$) and in the adsorbed layer ($\frac{\gamma_{p}^{a}}{\gamma_{oct}^{a}}$) as well as with the active site on the surface (K_s). UNIFAC

estimates for $\frac{\gamma_{oct}}{\gamma_p}$ predict that this ratio changes as $x_P^{-1.2}$. Numerical simulations in Fig. S4 show hypothetical dependencies of θ_p on x_p and $\frac{\gamma_p^a}{\gamma_{oct}^a}$.

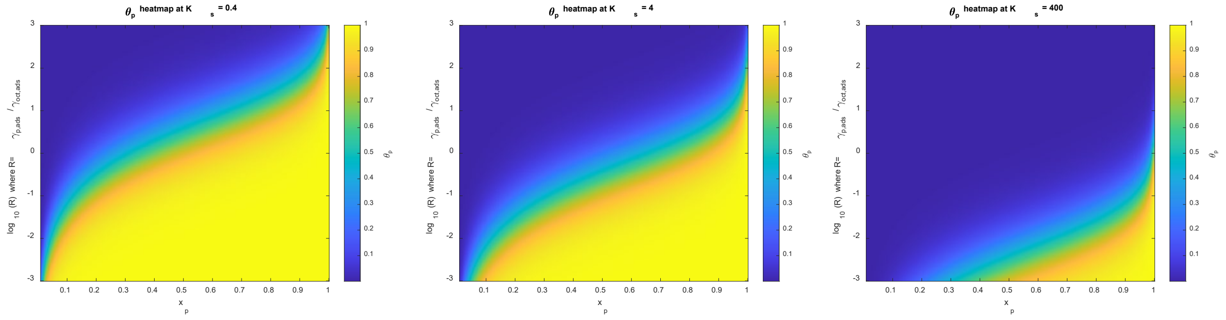

Fig. S4. Surface coverage of chemisorbed polymer according to eq. S11.

These qualitative results indicate that at relatively low K_s values corresponding to (-4)-6 kJ/mol change in energy (eq. S6, middle graph on Fig. S4), the surface will experience a relatively sharp transition from polymer-filled to octane-filled state. The exact shape of θ_p vs x_p curve will be determined by $\frac{\gamma_p^a}{\gamma_{oct}^a}$ dependence on x_p, which is generally unknown.

Experiments with n-octane and 1,4-dimethyl cyclohexane (Fig. S12) show that solvents with comparable K_s and $\frac{\gamma_{oct}}{\gamma_p}$ have different impacts on the polymer hydrogenolysis, likely due to non-idealities of chemisorbed layer ($\frac{\gamma_p^a}{\gamma_{oct}^a}$).

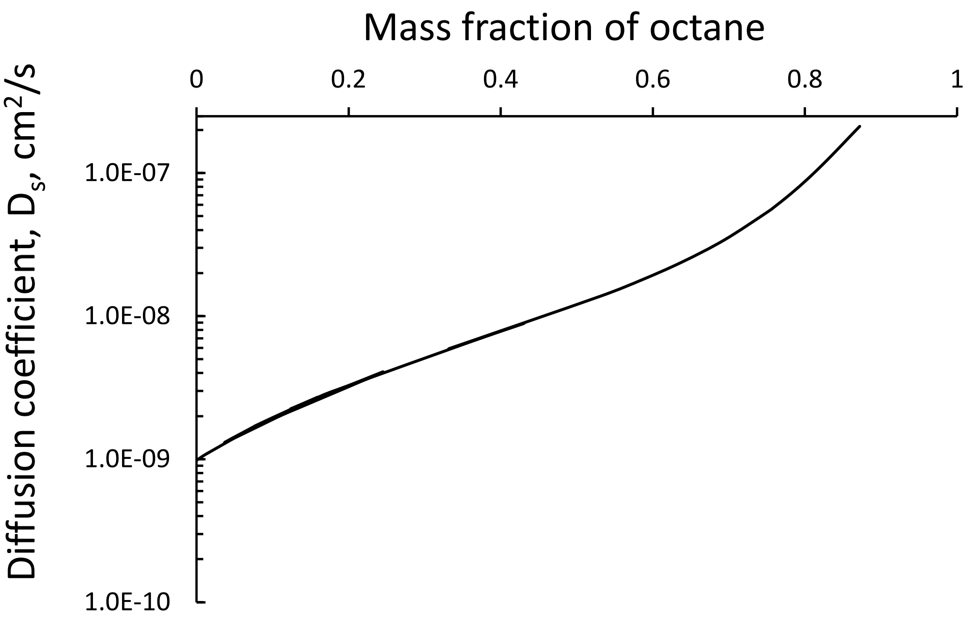

Fig. S5. HDPE self-diffusion coefficient at 250 °C as a function of octane mass fraction according to Von Meerwall et al.[4]

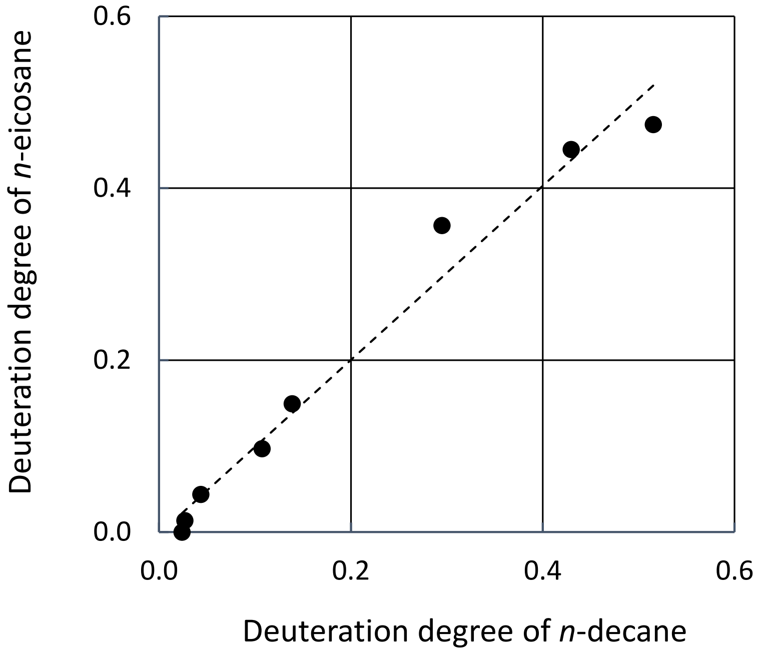

Fig. S6. Deuteration degree of eicosane *vs.* deuteration degree of *n*-decane. Conditions: 250 °C, 30 bar (H_2 or D_2), reaction time 10 min, 2 g HDPE, 50 mg Ru/TiO_2 catalyst, *n*-octane loading: 0-2.7 g.

The deuteriation degree (D_i) of different reaction products was calculated as follows:

$$D_i = \frac{\overline{D_i}}{2i+2},$$

wherein $\overline{D_i}$ is average number of deuterium atoms in the molecule, according to the distribution derived from the GC-MS, and $2i+2$ is the total amount of H or D atoms available for substitution in the alkane molecule with i carbons.

The linear correlation indicates that decane is a representative reaction product, and other products exhibit similar deuteration degrees.

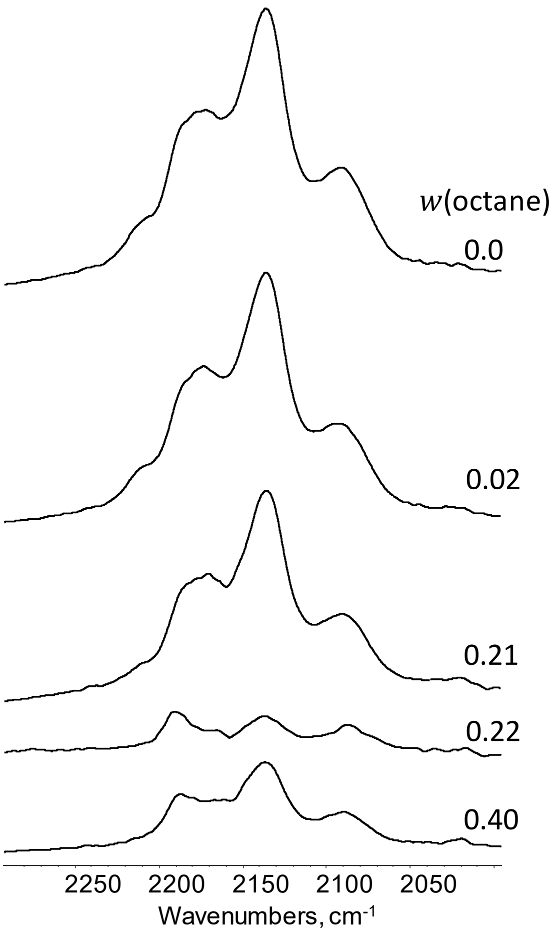

Fig. S7. – Attenuated total reflectance (ATR) Fourier transform infrared (FTIR) spectra in the C-D stretching region of deuterated solid residue. Conditions: 250 °C, 30 bar D_2, reaction time 10 min, 2 g HDPE, 50 mg Ru/TiO_2 catalyst, *n*-octane loading: 0-2.7 g.

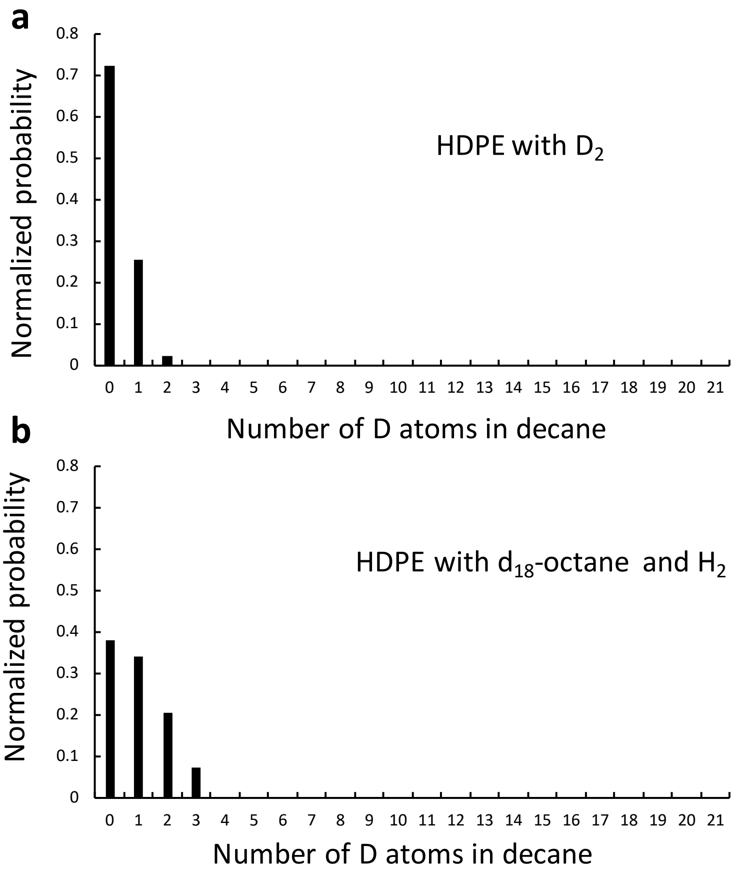

Fig. S8. Distribution of isotopomers in $C_{10}H_{22-x}D_x$ *n*-decane reaction product for two reaction mixtures: a) D_2 gas, 2 g HDPE, and 2.7 g *n*-octane C_8H_{18}, and b) H_2 gas, 2 g HDPE, 1.9 g C_8H_{18} and 0.9 g d_{18}-octane. Conditions: 30 bar H_2 (or D_2), 250 °C, 10 min.

Decane, as a representative reaction product, is deuterated by D_2, yielding a $C_{10}H_{21.70}D_{0.30}$ average composition. When the isotope label is introduced *via* the d_{18}-octane solvent, decane shows a shift in the distribution to ~1 atom being exchanged, leading to $C_{10}H_{21.03}D_{0.97}$ average composition.

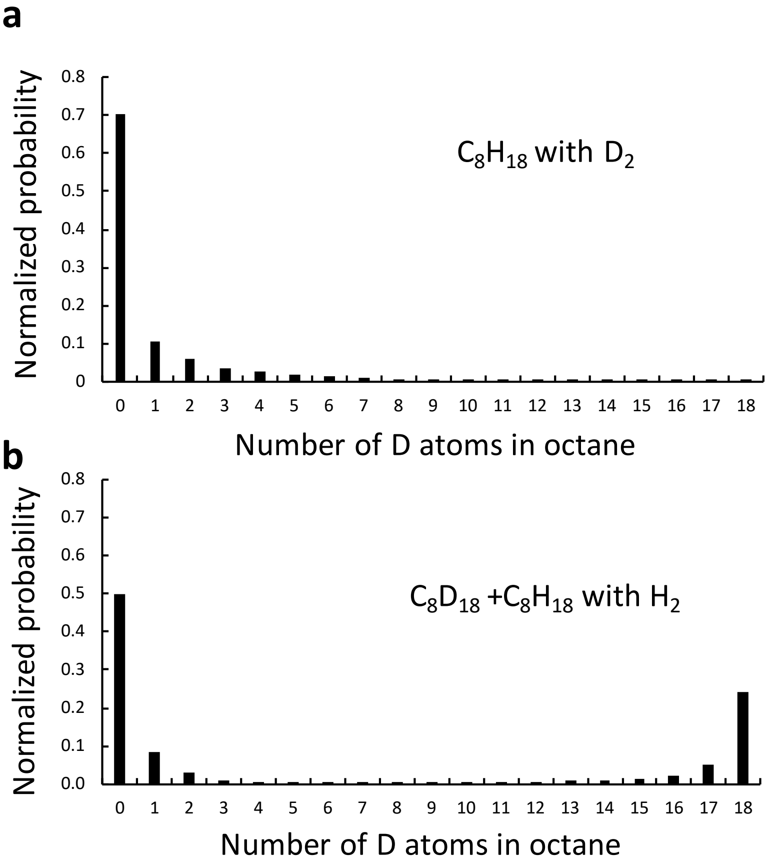

Fig. S9. Distribution of isotopomers in $C_8H_{18-x}D_x$ *n*-octane solvent for two reaction mixtures: a) D_2 gas, 2 g HDPE, and 2.7 g C_8H_{18} and b) H_2 gas, 2 g HDPE, 1.9 g C_8H_{18}, and 0.9 g d_{18}-octane. Conditions: 30 bar H_2 (or D_2), 250 °C, 10 min.

During the reaction of an HDPE–*n*-octane mixture in the presence of D_2, *n*-octane undergoes an exchange of 1–2 hydrogen atoms with deuterium atoms, resulting in an average composition of $C_8H_{16.97}D_{1.03}$. In the analogous reaction between H_2 and fully deuterated d_{18}-octane, a similar exchange of 1–2 atoms is observed. When the mixture of *n*-octane isotopomers (C_8H_{18} and C_8D_{18}) reacts with H_2, the isotopic distribution curve has two distinct peaks at 0 and 18 deuterium atoms, corresponding to the initial isotopomers of *n*-octane.

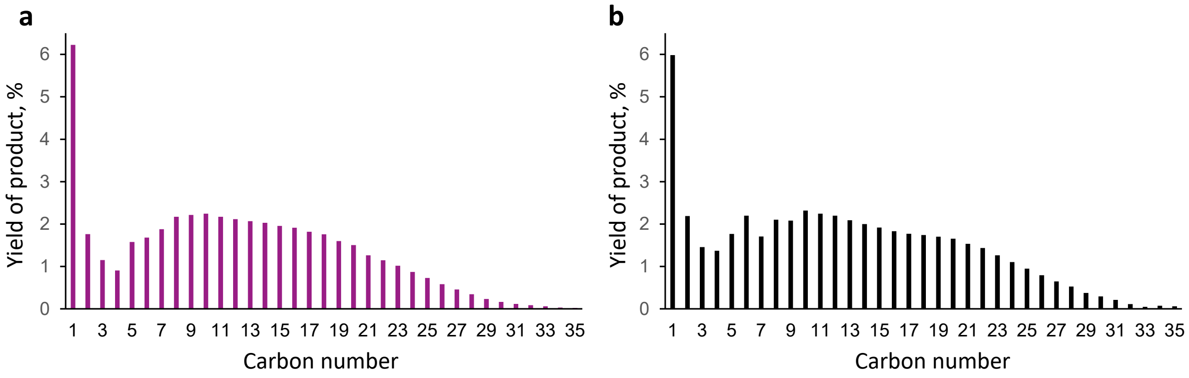

Fig. S10. Liquid and gas product distributions for fresh Ru/TiO_2 catalyst (a) and catalyst-treated in *n*-octane (b). Conditions: 30 bar H_2, 250 °C, 1 h, 2 g HDPE, 50 mg of fresh catalyst, and 35 mg of *n*-octane-treated catalyst.

Ru/TiO_2 was tested and filtered after reaction with *n*-octane at 250 °C for 2 h. The product distribution shows that the catalyst selectivity and activity patterns were not affected by the treatment with *n*-octane.

Table S3. Octane solvent conversion during HDPE hydrogenolysis.

n-Octane weight fraction	Octane conversion
-	-
0.05	6.3
0.10	8.0
0.16	10.9
0.19	11.5
0.20	8.1
0.21	6.8
0.22	5.0
0.40	6.9

Conditions: 250 °C, 30 bar H_2, 2 g HDPE, 1 h reaction time, 50 mg Ru/TiO_2 catalyst.

Octane conversion was estimated from the remaining amount of octane:

$$octane\ conversion = \frac{N_{C_8}^0 - \left(N_{C_8} - N_{C_9}\right)}{N_{C_8}^0},$$

Where $N_{C_8}^0$- initial number of moles of octane; $N_{C_8}$and N_{C_9} – number of moles of octane and nonane in the product mixture. This estimation assumes that nonane is selectively formed only from HDPE, while octane originates from added octane solvent and HDPE reactivity. We estimate octane formed from HDPE as being approximately equal to nonane formed from HDPE assuming smooth product distribution curves (Fig. S9).

Table S4. Effect of the chain length of the added alkane structure on HDPE hydrogenolysis.

Additive	*n*-Alkane weight fraction in the melt	Reaction time, h	Yield of extractables, %
none	-	1	59.4
n-octane	0.05	1	65.3
n-hexadecane	0.05	1.2	47.3
n-tetracosane	0.05	1.4	27.0

Conditions: 250 °C, 30 bar H_2, 2 g HDPE, 50 mg Ru/TiO_2 catalyst.

Table S5. Effect of *n*-octane addition on HDPE hydrogenolysis.

n-Octane weight fraction	Reaction time, h	Carbon yields, %			M_n, g/mol[1]
		Methane	Total extractables	Solid residue	
0	0.5	5.1	25.9	66.9	11200
	1	6.2	47.9	40.6	600
	2	8.0	97.3	3.5	-
0.02	0.5	3.5	19.1	57.7	2100
	1	4.5	70.0	33.9	1000
0.05	0.5	2.9	14.5	72.6	-
	1	5.0	65.3	34.5	1300
0.40	1	3.0	11.8	86.0	-
	2	2.4	16.0	84.0	3100
	3.3	6.5	56.7	43.4	-

Conditions: 250 °C, 30 bar H_2, 2 g HDPE, 50 mg Ru/TiO_2 catalyst.

[1] Number-average molecular weight of solid residue, $M_n = \frac{\sum \frac{dw}{dlogM}}{\sum \frac{dw}{dlogM} \cdot \frac{1}{M}}$. [2]

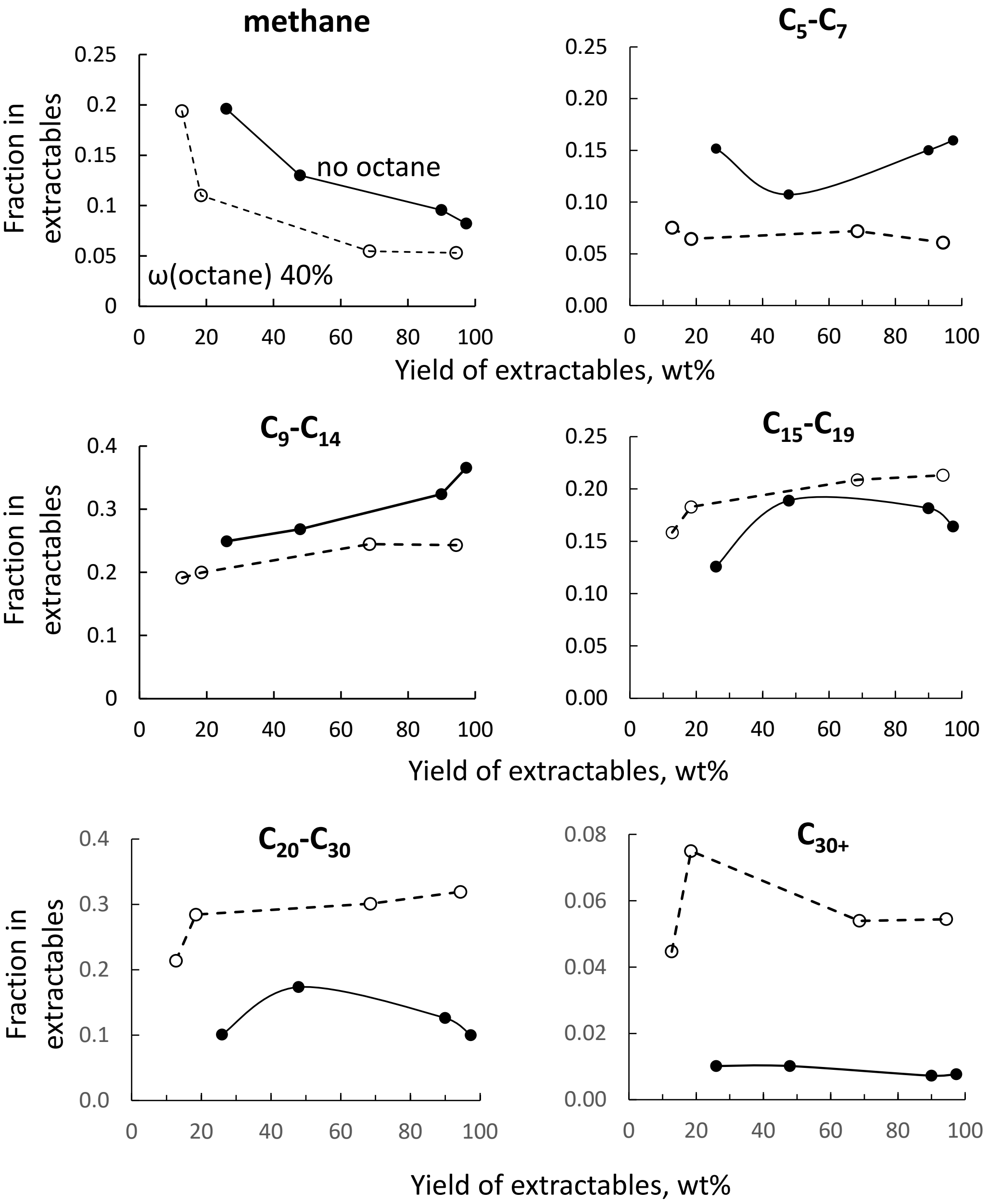

Fig. S11. Effect of *n*-octane addition on product group selectivity as a function of the yield of extractables. Solid lines - data without *n*-octane, dashed lines – with 2.7 g of octane. Conditions: 250 °C, 30 bar H_2, 2 g HDPE, 50 mg Ru/TiO_2 catalyst, 0 or 2.7 g *n*-octane added.

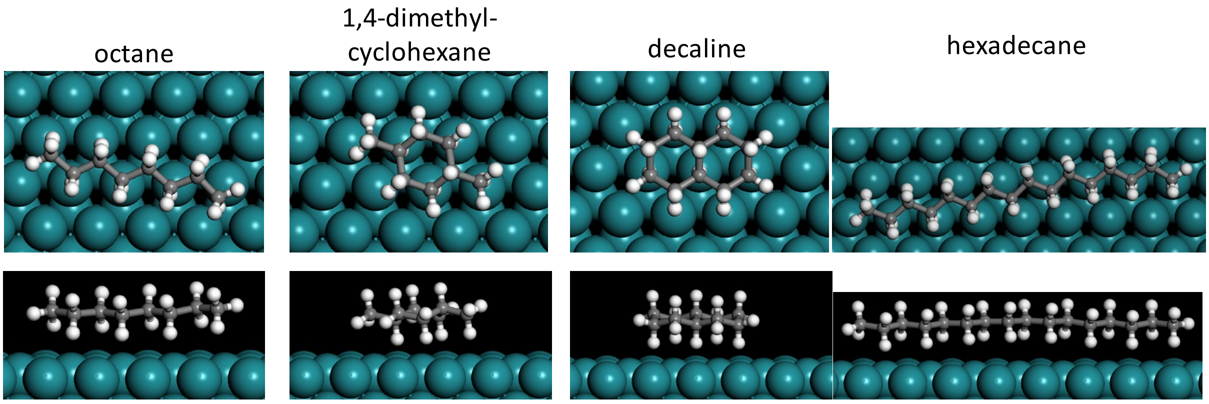

Fig. S12. Top and side views of DFT-optimized structures of physisorbed alkane solvents on Ru (0001) surface.

References

(1) Balzer, A. H.; Hinton, Z. R.; Vance, B. C.; Vlachos, D. G.; Korley, L. T.; Epps III, T. H. Tracking Chain Populations and Branching Structure during Polyethylene Deconstruction Processes. *ACS Central Science* **2024**, *10* (9), 1755-1764.
(2) Evans, J. Gel permeation chromatography: a guide to data interpretation. *Polymer Engineering & Science* **1973**, *13* (6), 401-408.
(3) Semenchenko V.K. Surface Phenomena in metals and alloys. Gostechtheorizdat, USSR, 1957.
(4) Von Meerwall, E.; Feick, E.; Ozisik, R.; Mattice, W. Diffusion in binary liquid n-alkane and alkane-polyethylene blends. *The Journal of Chemical Physics* **1999**, *111* (2), 750-757.
